# Organ dysfunction during continuous veno-venous high cut-off hemodialysis in patients with septic acute kidney injury: A prospective observational study

**DOI:** 10.1371/journal.pone.0172039

**Published:** 2017-02-16

**Authors:** Gianluca Villa, Cosimo Chelazzi, Elena Morettini, Lucia Zamidei, Serafina Valente, A. Lucia Caldini, Giovanni Zagli, A. Raffaele De Gaudio, Stefano Romagnoli

**Affiliations:** 1 Department of Health Science, Section of Anesthesiology and Intensive Care, University of Florence, Florence, Italy; 2 Department of Anesthesia and Intensive Care, Azienda Ospedaliero-Universitaria Careggi, Florence, Italy; 3 Anesthesia and Intensive Care Unit, Ospedale Santo Stefano, Prato, Italy; 4 Intensive Cardiac Coronary Unit, Heart and Vessel Department, Azienda Ospedaliero-Universitaria Careggi, Florence, Italy; 5 Clinical Chemistry Laboratory, Azienda Ospedaliero-Universitaria Careggi, Florence, Italy; Bambino Gesù Children's Hospital, ITALY

## Abstract

**Background:**

Continuous veno-venous hemodialysis with high cut-off membranes (HCO-CVVHD) removes inflammatory mediators involved in organ dysfunction during sepsis. The aim of the present study was to assess the variations in SOFA score and identify early predictors of short-term mortality in a cohort of patients with septic shock, treated with HCO-CVVHD for acute kidney injury (AKI).

**Methods:**

An observational prospective multicenter cohort study was conducted in four mixed medical-surgical ICUs. Thirty-eight patients with septic shock and AKI (KDIGO stage≥1) treated with HCO-CVVHD have been included in this study. Patients were divided into Survivors and non-Survivors according to mortality observed at 72^nd^ hr of treatment. The variation of SOFA scores and clinical/biochemical parameters were described over time for the entire population and specifically for Survivors and non-Survivors. Similarly, circulating inflammatory mediators (as IL-6, TNF-a and IL-10) were described over time. A logistic regression analysis was used to identify the baseline clinical and biochemical parameters associated with 72 hrs-ICU mortality.

**Results:**

Overall, the mean SOFA score was 12±3 at baseline, 10.9±3 at 6hrs, 9.8±3 at 12hrs, 8.9±3.3 at 24 hrs, and 8±3.5 at 48 hrs after HCO-CVVHD initiation; and 6.5±2.7 at 24 hrs and 6.6±3 at 48 hrs after HCO-CVVHD discontinuation. In the multivariate regression analysis, baseline serum lactate levels and AKI stage independently correlated with short-term mortality during HCO-CVVHD. A significant reduction was observed in circulating levels of TNFα and IL-6 among Survivors.

**Conclusions:**

SOFA score significantly decreased early after initiation of HCO-CVVHD in patients with septic AKI. Baseline lactate levels and the AKI stage resulted to be associated to 72 hrs-ICU-mortality.

## Introduction

Severe sepsis and septic shock are common causes of death among patients admitted to intensive care units (ICUs) [1,2]. Acute kidney injury (AKI) is an independent risk factor for death in these patients [3]. Persistent systemic inflammation, immunoparalysis and secondary infections are thought to play key roles in sepsis-related organ dysfunction [4]; indeed, an imbalance between pro- (e.g. IL-1 and 6, TNFα) and anti-inflammatory (e.g. IL-4 and 10) cytokines may be associated with a worse clinical outcome [5]. Unselective removal of inflammatory and anti-inflammatory mediators by means of blood purification therapies may positively impact organ dysfunction in septic patients, particularly regarding hemodynamic and respiratory functions [6]. As such, continuous veno-venous hemodialysis with high cut-off membrane (HCO-CVVHD) has been used as an adjuvant therapy in septic patients with AKI [7]. The higher-than-standard pore size (i.e. >0.01μm) allows HCO to remove large molecules such as inflammatory mediators [6]. The aim of this observational study was to describe the variations in Sequential Organ Failure Assessment (SOFA) scores, and to identify baseline clinical and biochemical parameters statistically associated with early mortality in a cohort of patients with septic shock, treated with HCO-CVVHD for AKI.

## Material and methods

This was an observational prospective multicenter study performed in four mixed general ICUs. The regional Ethical Board (“Comitato Etico di Area Vasta Centro, Regione Toscana”) reviewed and approved the protocol for all participating centers (n° 2013/0024940). The consent for analysis and publication of clinical data have been written obtained by the patients, if conscious. If the patient was not able to sign consent forms at the study enrollment, permission for analysis and publication of clinical data was obtained before the patient's ICU discharge (for survived patients) or it was waived (for dead patients) in accordance with local ethics committee. Consent forms are stored in the ICU of the principal investigator (Prof. A. R De Gaudio).

**Fig 1:**
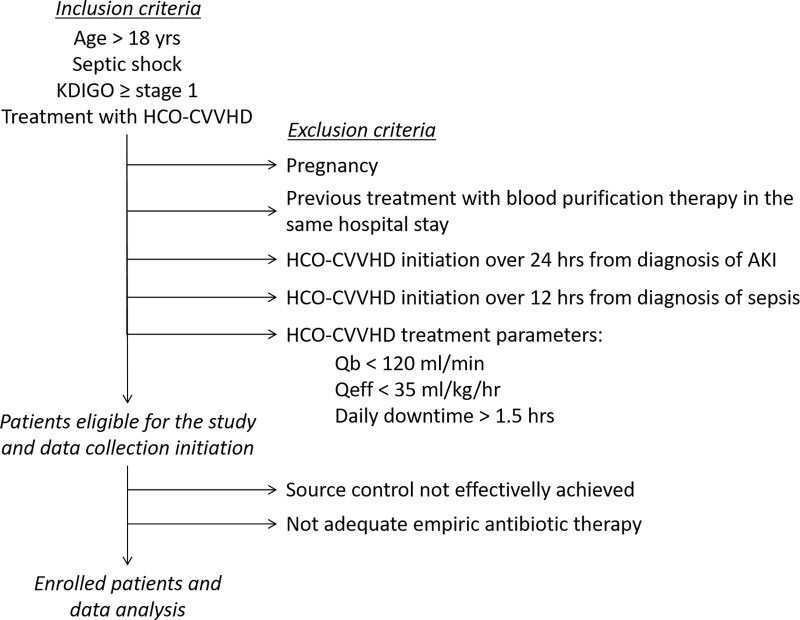
Inclusion and exclusion criteria.

All patients with septic shock (defined according to the Surviving Sepsis Campaign guidelines [8]) and AKI (defined as a KDIGO stage≥1 [9]) undergoing HCO-CVVHD were considered for inclusion. Inclusion and exclusion criteria are described in Fig 1. HCO-CVVHD was performed using a Prismaflex machine (Gambro Lundia AB–Lund, Sweeden) and SepteX membranes (a polyarylethersulfone high cut-off membrane with a 1.1m2 surface area; Gambro Lundia AB–Lund, Sweeden) in all patients. Treatment parameters prescribed and actually delivered during HCO-CVVHD have been derived from memory cards within Prismaflex machines used. Clinical and biochemical observations have been limited at the first 72 hrs of treatment with HCO-CVVHD, i.e. the maximum time of treatment achievable with a single extracorporeal circuit according to the filter manufacturer. According to local routine clinical practice, HCO-CVVHD discontinuation has been decided according to recovery of clinical stability (mostly in terms of hemodynamic status) and renal function. In particular, at the end of the HCO filter life-span, if patient was clinically improved and able to maintain an adequate fluid balance/urinary flow/solute control (spontaneously or with diuretic administration), she or he was considered adequately weaned from HCO-CVVHD. On the other hand, if clinical stability was not associated with recovery of good renal function at the end of HCO life-span, HCO-CVVHD was substituted with another CRRT modality performed with a high-flux membrane (usually CVVHDF).

For sample size evaluation, the mean SOFA score in patients with AKI and sepsis, at baseline and during the following four days, was derived from the literature [10]. Assuming a mean mortality rate of 30% during the first 48 hrs of treatment, a sample size of 38 patients was needed to observe a reduction in SOFA score equal to 2.5 at 48 hrs after HCO-CVVHD initiation through a t-Student test for paired data with a power of 90% and a type I error rate of 2.5%. The SOFA score reduction equal to 2.5 has been chosen considering this as the minimal SOFA score variation with a clinical relevance.

Patients have been divided in “Survivors” and “non-Survivors” groups according to the occurrence of death during the 72 hrs of HCO-CVVHD. SOFA score, Acute Physiology and Chronic Health Evaluation (APACHE) II, clinical and biochemical data were recorded at baseline, then at 6, 12, 24, and 48 hrs after HCO-CVVHD [11] initiation; and at 24 and 48 hrs after HCO-CVVHD discontinuation. If another HCO-CVVHD treatment was required after these first 72 hrs, the latter time points were calculated from the discontinuation of the last HCO filter utilized. All parameters were described both for Survivors and non-Survivors groups. In addition, the circulating levels of inflammatory mediators (IL-6, TNF-a and IL-10) were measured at baseline, then at 24 and 48 hrs after HCO-CVVHD initiation, and at 24 hrs after HCO-CVVHD discontinuation.

The Shapiro-Wilk test was used to test the data for normality. Continuous variables were presented as mean ± standard deviation (SD) or median [I-III interquartile range] where appropriate. The variations of these parameters over time with respect to the baseline were evaluated using t-Student test for paired data or Wilcoxon signed-rank test, according to data distribution. Similarly, the differences between Survivors and not- Survivors were tested using t-Student test for un-paired data or Mann-Whitney Wilcoxon. Categorical variables were analyzed using the chi-square test and given as a percentage. For each time point, a mean difference (SD) was expressed to evaluate the SOFA score trend in the observed population with respect to the baseline value. Finally, the differences in baseline characteristics between groups were analyzed through univariate and multivariate regression analysis in order to identify the baseline parameters associated with survival at 72 hrs of treatment. All variables with a p value less than 0.3 at the univariate analysis have been considered for the multivariable analysis. Statistical results were expressed as *p* value, Odds Ratio (OR) and 95% confidence interval (95%CI). For continuous variables statistically associated with survival at 72 hrs of treatment at multivariate analysis, a ROC analysis was performed in order to analyze its accuracy in predicting outcome and to identify a cut-off value applicable in clinical practice.

Data were analyzed using STATA 9.1 software (STATA corp, 490, Lakeway Drive College Station, 77845, Texas, US).

## Results

The study included 38 patients (S1 Table) enrolled from March 2013 to September 2014. Of these, 17 (44.7%) were medical and 21 (55.2%) were surgical. Table 1 summarizes baseline patients’ clinical characteristics for the entire population and specifically for Survivors (N = 30) and non-Survivors (N = 8). Prescribed and delivered treatment parameters for both groups, calculated according to standard nomenclature [12,13], are described in Table 2. Excluding a transient hypotension at the HCO-CVVHD initiation that occurred in 5 patients, no others adverse events have been reported during treatments. All hypotensive episodes have been mostly attributed to the blood filling into the extracorporeal circuit; they did not require further increase of vasoactive drugs and spontaneously recovered. Exogenous replacement for albumin or coagulation factors were necessary for none of the observed patients during treatments.

**Table 1:** Baseline characteristics of the overall population and for the separate subgroups of Survivors and not- Survivors.

Variables	Tot	Survivors (N = 30)	non-Survivors (N = 8)	*p*	OR	95%CI
**Male gender**	71.1%	73.3%	62.5%	0.55	0.60	0.11–3.14
**Age (years)**	74.5 [64–79]	75 [64–80]	72 [68.5–77]	0.76	1.01	0.94–1.09
**Microorganisms**						
*Klebsiella spp*.	21.1%	20%	25%	0.43	2.99	0.20–44.73
*E*. *coli*	21.1%	20%	25%	0.36	4.32	0.18–97.53
*Ps*. *aeruginosa*	21.1%	20%	25%	0.37	3.80	0.21–69.85
*St*. *aureus*	21.1%	20%	25%	0.37	3.22	0.25–41.39
*Str*. *pneumoniae*	10.5%	10%	12.5%	0.45	3.22	0.16–68.19
*Candida spp*.	21.1%	20%	25%	0.91	1.14	0.14–9.53
*Aspergillum spp*.	10.5%	10%	12.5%	0.97	1.06	0.05–23.83
*Other*	18.4%	20%	12.5%	0.84	0.74	0.01–0.90
**Source of infection**						
*Pneumonia*	31.6%	30%	37.5%	0.69	1.4	0.27–7.15
*Peritonitis*	23.7%	26.7%	12.5%	0.42	0.39	0.04–3.71
*Urinary tract*	10.5%	6.7%	25%	0.16	4.67	0.54–40.03
*Blood stream*	18.4%	16.7%	25%	0.59	1.67	0.26–10.77
*Soft tissue*	15.8%	16.7%	12.5%	0.78	0.71	0.07–7.16
**APACHE II**	37.55±3.55	37.6±3.06	38±2.92	0.89	0.99	0.89–1.11
**SOFA**	12.02±3.01	12.10±2.99	11.75±3.33	0.77	0.96	0.74–1.25
**KDIGO**				0.05	8.03	0.99–64.8
*Stage 1*	15.8%	20%	0%			
*Stage 2*	34.2%	40%	12.5%			
*Stage 3*	50%	40%	87.5%			
**Norepinephrine (mcg/kg/min)**	0.20 [0.15–0.30]	0.23 [0.15–0.30]	0.18 [0.08–0.33]	0.77	0.38	0.0–292.57
**SAP (mmHg)**	103±27	105±26	95±27	0.34	0.98	0.95–1.01
**MAP (mmHg)**	65±15	67±15	60±14	0.24	0.96	0.91–1.03
**Heart rate (bpm)**	83 [80–100]	83 [80–99]	88 [80–106]	0.37	1.02	0.98–1.06
**CVP (mmHg)**	12±4	11±4	13±5	0.32	1.11	0.91–1.36
**RR (breath/min)**	18±6	18±6	18±5	0.89	0.99	0.86–1.14
**PaO**_**2**_**/FiO**_**2**_	230±126	227±117	240±126	0.79	1.00	0.99–1.00
**PEEP (cmH2O)**	6 [5–8]	6 [4–8]	7 [6–8]	0.36	1.15	0.86–1.53
**Arterial HCO3- (mmol/L)**	22.3±3.9	22.9±3.8	19.8±3.4	0.05	0.75	0.56–0.99
**Lactate (mmol/L)**	2.5 [1.5–4.8]	2.4 [1.4–3.3]	6.3 [3.7–14.3]	0.01	1.35	1.06–1.71
**ScvO**_**2**_ **(%)**	58±15	60±15	55±14	0.44	0.98	0.92–1.04
**WBC (10**^**9**^**/L)**	17.89±11.72	18.20±12.17	16.73±10.53	0.75	0.99	0.92–1.06
**Plt (10**^**9**^**/L)**	97 [67–226]	100 [68–226]	83[33.5–206.5]	0.78	1.00	0.99–1.00
**AT (%)**	60±21	62±21	54±23	0.38	0.98	0.94–1.02
**PCT (ng/ml)**	5.6 [2–20.4]	4.5 [2–20.6]	6.4 [3.7–7.2]	0.34	0.97	0.90–1.03
**CRP (mg/L)**	143[65–237]	143[45–248]	143[81–187]	0.81	1.00	0.99–1.01

Differences between groups were obtained using univariate logistic regression analysis. Abbreviations: SAP, systolic arterial pressure; MAP, mean arterial pressure; CVP, central venous pressure; RR, respiratory rate; PEEP, positive end expiratory pressure; ScvO_2_, central venous oxygen saturation; WBC, white blood cells; Plt, platelets; AT, antithrombin; PCT, procalcitonin; CRP, C-reactive protein.

**Table 2:** HCO-CVVHD treatment parameters across the groups.

	Tot	Survivors (N = 30)	Non-Survivors (N = 8)	*p*	OR	95%CI
**Weight (Kg)**	71 [67; 78]	71 [66; 78]	72 [70; 77.5]	0.53	1.05	0.91–1.21
**Precribed dose (ml/kg/hr)**	35	35	35			
**Qb (ml/min)**	132.5 [125;143]	132.5 [125; 140]	132 [124; 147.5]	0.72	1.01	0.93–1.10
**Qd (ml/hr)**	2350 [2214; 2587]	2280 [2094; 2511]	2388 [2228; 2448]	0.76	1.00	0.99–1.00
**NET UF (ml/hr)**	73.5 [61; 86]	73.5 [62; 87]	74 [56.5; 86]	0.74	0.99	0.94–1.04
**Downtime (min)**	86.6 [38; 124.9]	80 [24.9; 125.5]	91.8 [60.9; 121.8]	0.63	1.00	0.99–1.01
**Delivered dose (ml/kg/hr)**	32.89 [31.96; 34.07]	33.05 [31.95; 34.39]	32.77 [32.04; 33.52]	0.62	0.86	0.47–1.57
**Delivered/Prescribed dose (%)**	93.9 [91.3; 97.4]	94.4 [91.3; 98.3]	93.6 [91.5; 95.8]	0.63	0.95	0.77–1.17
**Anticoagulation**				0.63	1.00	0.98–1.01
*Heparin*	17 (44.7%)	13 (43.3%)	4 (50%)			
*No-Anticoagulation*	21 (55.3%)	17 (56.7%)	4 (50%)			
**CRRT requirements after 72 hrs**	12 (40%)	12 (40%)	-			
*No-CRRT*	18 (60%)	18 (60%)				
*HCO-CVVHD*	2 (6.7%)	2 (6.7%)				
*HF-CVVHDF*	10 (33.3%)	10 (33.3%)				

Patients have been treated without anticoagulation to reduce risk of bleeding after surgical source control. Differences between groups were obtained using univariate logistic regression analysis. Abbreviations: Qb, Blood flow; Qd, Dialysate flow; NET UF, Net ultrafiltration, HF-CVVHDF, Hemodiafiltration with high-flux membrane.

**Fig 2:**
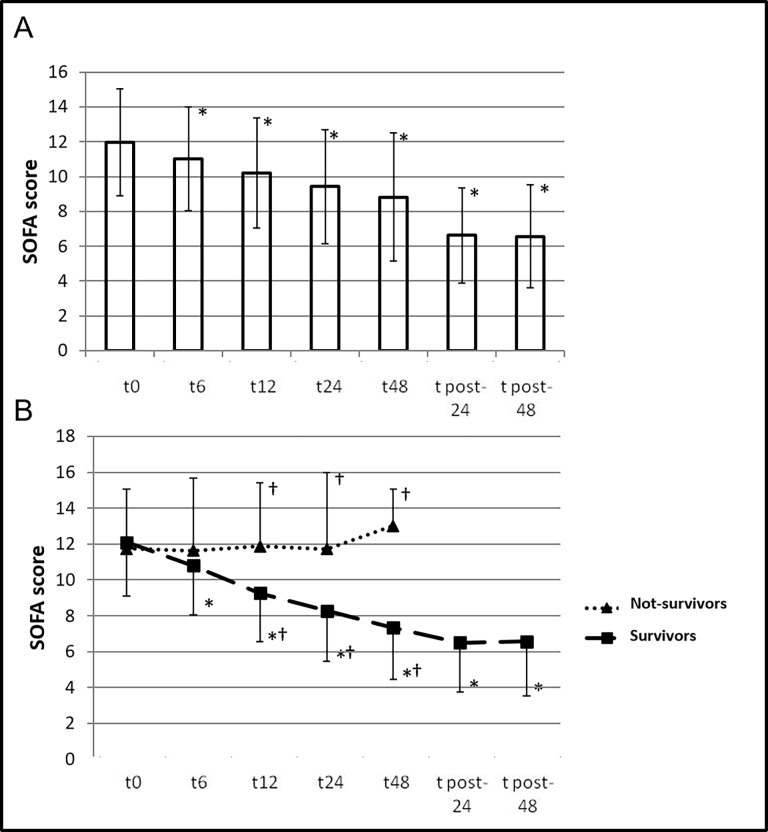
SOFA score. Mean and SD of SOFA scores cumulatively observed for the entire population at the baseline, and then at 6, 12, 24 and 48 hrs after HCO-CVVHD initiation; and at 24 and 48 hrs after HCO-CVVHD discontinuation (Panel A). In Panel B, SOFA scores are shown for each subgroup (Survivors and non-Survivors). * Value statistically different to the baseline through statistical test for pairs data. † Value statistically different between groups through statistical test for unpaired data.

**Fig 3:**
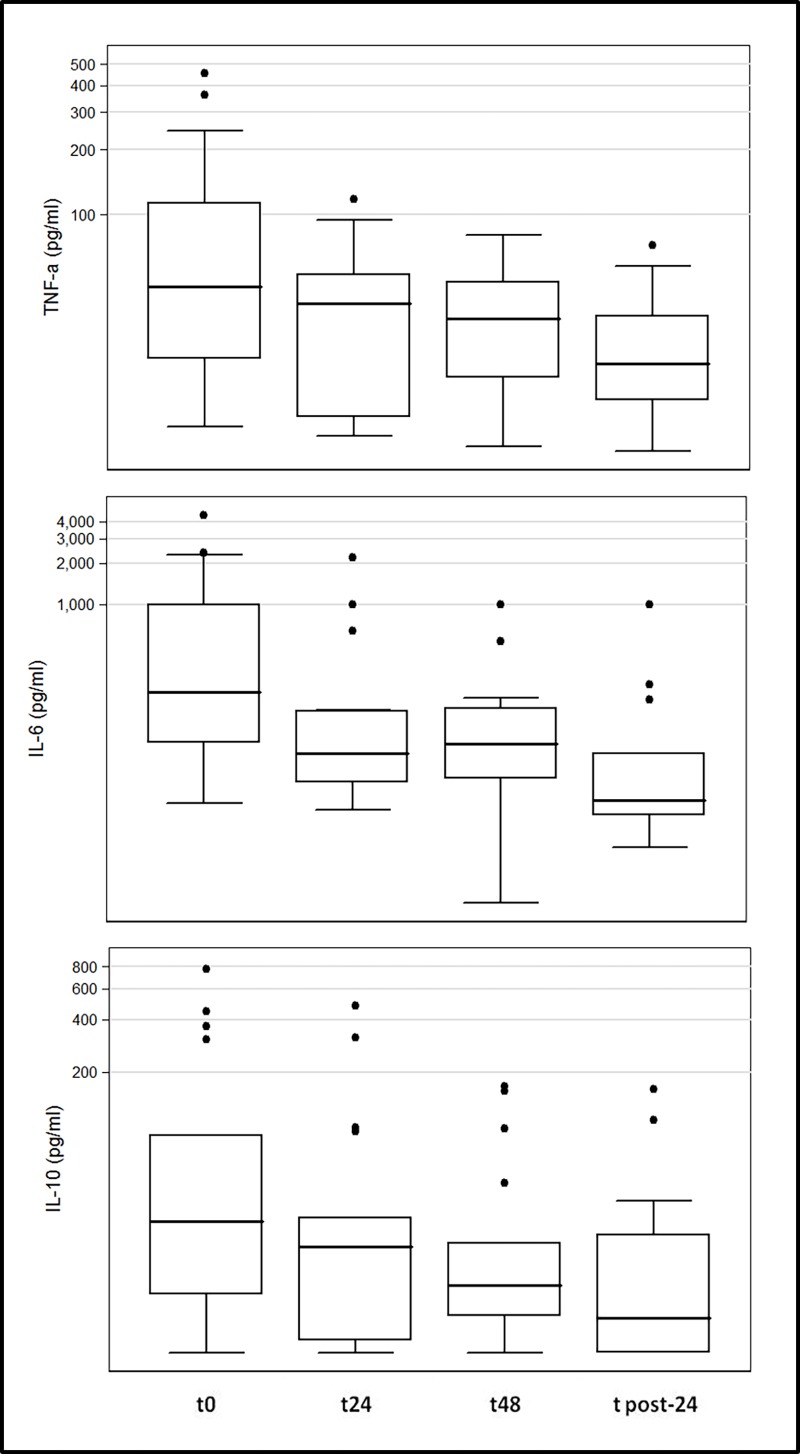
Circulating levels of inflammatory mediators. Concentrations of mediators are expressed in a logarithmic scale.

At the HCO-CVVHD initiation (baseline), the mean SOFA score was 12.03±3.02. During treatment, the mean±SD values of SOFA score were 10.97±3 at 6 hrs (N = 38; *p*<0.001; mean difference 1.05±1.45, 95%CI[0.58–1.53]), 9.82±3.03 at 12 hrs (N = 38; *p*<0.001; mean difference 2.21±1.99, 95%CI[1.56–2.86]), 8.91±3.34 at 24 hrs (N = 37; *p*<0.001; mean difference 3.03±2.72, 95%CI[2.12–3.93]), and 8±3.48 at 48 hrs after HCO-CVVHD initiation (N = 34; *p*<0.001; mean difference 4.06±3.48, 95%CI[2.84–5.27]). After HCO-CVVHD discontinuation, SOFA scores were 6.5±2.73 at 24 hrs (N = 30; *p*<0.001; mean difference 5.42±3.78, 95%CI [3.96–6.90]) and 6.57±3.04 at 48 hrs (N = 30; *p*<0.001; mean difference 5.36±3.99, 95%CI [3.80–6.90]). Fig 2 shows the variation of SOFA score over time for the entire population and specifically for Survivors and non-Survivors groups. Clinical parameters observed for Survivors and non-Survivors at each time point are presented in Table 3. TNFα and IL-6 significantly decreased during the treatment, particularly for Survivors (p<0.05; see Table 3 and Fig 3). At multivariate logistic regression analysis the baseline serum lactate (*p* = 0.03, OR 1.45, 95%CI [1.01–2.06]) and initial KDIGO stage (*p* = 0.04, OR 7.79, 95%CI[0.93–65.4]) were independently associated to 72 hrs-ICU mortality. A ROC analysis for baseline lactate levels has shown a ROC-AUC equal to 0.79 (95%CI, 0.57–1.00). Sensitivity and specificity related to the different baseline lactate levels were calculated. With a sensitivity equals to 75% and a specificity equals to 90%, a baseline lactate level of 5.2 mmol/l was identified as the cut-off value that best distinguishes between patients who survived and those who died after 72 hrs of HCO-CVVHD. Every patient not-survived during this observational study dead during HCO-CVVHD. The cumulative hospital mortality rate for the entire cohort was 39.5%.

**Table 3:** Clinical and biochemical parameters for both the overall population and the Survivors and non-Survivors subgroups.

Variables	t0 (baseline)	t6	t12	t24	t48	t post-24	t post-48
**Sample size (pts)**	38	38	38	37	34	30	30
Survivors	30	30	30	30	30	30	30
Non-Survivors	8	8	8	7	4	-	-
**Norepinephrine (mcg/kg/min)**	0.20 [0.15–0.30]	0.20 [0.10–0.30]	0.20 [0.05–0.25]*	0.10 [0–0.23]*	0.10 [0–0.20]*	0.05 [0–0.10]*	0 [0–0.10]*
Survivors	0.23 [0.15–0.30]	0.18 [0.10–0.30]*	0.18 [0–0.25]*	0.08 [0–0.15]*^†^	0.06 [0–0.10]*^†^	0.05 [0–0.10]*	0 [0–0.10]*
Non-Survivors	0.18 [0.08–0.33]	0.20 [0.13–0.25]	0.20 [0.09–0.25]	0.20 [0.20–0.30]^†^	0.20 [0.20–0.25]^†^	-	-
**SAP (mmHg)**	103±27	106±20	110±24	106±26	117±27*	123±23*	121±22*
Survivors	105±26	110±18^†^	115±23^†^	112±17^†^	121±24*^†^	123±23*	121±22*
Non-Survivors	95±27	91±20^†^	92±17	79±36^†^	83±28^†^	-	-
**PAM (mmHg)**	65±15	68±13	69±12	69±14	72±16*	71±18	73±13
Survivors	67±15	71±12*^†^	71±12*^†^	71±13	75±14*^†^	70±18^#^	72±13
Non-Survivors	60±14	56±13^†^	59±10^†^	62±18	50±17^†^	-	-
**HR (bpm)**	83 [80–100]	87 [80–95]	83 [76–97]	80 [75–90]*	79 [70–88]*	80 [70–90]*	80 [70–90]*
Survivors	83 [80–99]	88 [78–95]	80 [76–95]	80 [74–85]*	77 [70–85]*	80 [70–90]*	80 [70–90]*
Non-Survivors	88 [80–106]	82 [80–102]	94 [80–106]	91 [75–107]	90 [75–90]	-	-
**CVP (mmHg)**	12±4	13±3	12±4	13±4	12±4	11±4	11±4
Survivors	11±4	12±3	11±4	12±4	12±4	11±3	11±3
Non-Survivors	13±5	14±2	12±3	14±4	13±3	-	-
**RR (breath/min)**	18±6	17±5	16±5	16±5	17±5	18±5	18±5
Survivors	18±6	17±5	15±5*	16±5	17±6	18±5	18±5
Non-Survivors	18±5	18±4	18±3	19±5	17±3	-	-
**PaO2/FiO2**	230±126	246±101	243±101	266±106	268±128	308±109*	297±110*
Survivors	227±117	247±92	247±96	276±101*	276±130*	308±109*	297±110*
Non-Survivors	241±166	242±137	229±123	222±120	209±115*	-	-
**Urinary output (ml/h)**	40 [0–80]	15 [0–50]*	18 [0–60]	10 [0–50]	20 [0–50]	83 [10–100]*	100 [20–150]*
Survivors	50 [0–80]	20 [0–50] ^†^	40 [10–60] ^†^	20 [0–50]	20 [0–50] ^†^	83 [10–100]*	100 [20–150]*
Non-Survivors	10 [0–70]	0 [0–0] ^†^	0 [0–10] ^†^	0 [0–20]	0 [0–0] ^†^	**-**	-
**Serum creatinine (mg/dl)**	1.8 [1.3–2.6]	1.5 [1.1–1.9]*	1.3 [0.9–1.5]*	1.1 [0.8–1.5]*	0.8 [0.6–1.2]*	1.2 [0.7–1.6]*	1.2 [0.7–1.9]*
Survivors	1.9 [1.4–2.8]	1.7 [1.1–2]* ^†^	1.3 [1–1.8]* ^†^	1.1 [0.8–1.6]*	0.8 [0.7–1.3]*	1.2 [0.7–1.6]*	1.2 [0.7–1.9]*
Non-Survivors	1.4 [1.3–1.5]	1.1 [1–1.4] ^†^	1 [0.8–1.3]* ^†^	0.9 [0.8–1.3]*	0.7 [0.4–1.1]	-	-
**Urea (mg/dl)**	0.8 [0.6–1.4]	0.7 [0.5–1.1]*	0.6 [0.4–0.9]*	0.5 [0.4–0.8]*	0.3 [0.3–0.5]*	0.6 [0.4–0.8]*	0.7 [0.3–1.1]*
Survivors	0.9 [0.6–1.7]	0.7 [0.4–1.2]*	0.7 [0.4–0.9]*	0.5 [0.4–0.8]*	0.4 [0.3–0.7]*	0.6 [0.4–0.8]*	0.7 [0.3–1.1]*
Non-Survivors	0.7 [0.4–0.8]	0.6 [0.5–0.8]	0.5 [0.3–0.7]	0.4 [0.3–0.6]	0.3 [0.2–0.4]	-	-
**Lactate (mmol/L)**	2.5 [1.5–4.8]	2.2 [1.7–5.1]	2 [1.3–3.4]*	1.9 [1.2–3]*	1.7 [1–2.4]*	1.3 [0.8–1.7]*	1 [0.7–1.6]*
Survivors	2.4 [1.4–3.3] ^†^	1.9 [1.7–4.1] ^†^	1.7 [1.2–2.8]* ^†^	1.6 [1–2.1]* ^†^	1.7 [1–2]* ^†^	1.3 [0.8–1.7]*	1 [0.7–1.6]*
Non-Survivors	6.3 [3.7–14.3] ^†^	6 [3.2–14.2] ^†^	6.5 [2.3–14.7] ^†^	3 [2.6–13.7] ^†^	10.7 [2.9–15] ^†^	-	-
**ScvO2 (%)**	58±14	65±12*	66±11*	67±11*	69±7*	72±6*	76±6*
Survivors	60±15	68±10*^†^	70±8*^†^	71±7*^†^	69±7*	72±6*	76±6*
Non-Survivors	53±14	52±11^†^	53±11^†^	51±11^†^	62±8*	-	-
**WBC (10**^**9**^**/L)**	15.8 [8–27.2]	16.4 [10.2–29.6]	14.7 [8.8–26.4]	13.9 [9.1–23.4]	13.3[7.8–22.4]	12.4 [7.5–16.8]*	13.2 [6.9–16.6]*
Survivors	16.2 [8–27.2]	15.6 [10.2–29.9]	14.6 [8.8–26.4]	14.5 [9.1–27.6]	13.7 [9.8–22.8]	12.4 [7.5–16.8]*	13.2 [6.9–16.6]*
Non-Survivors	15 [9.8–26.7]	20.6 [11.6–25.5]	17.6 [10.3–26.2]	12 [3.8–22.8]	8.1 [2.7–15.2]	-	-
**Plt (10**^**9**^**/L)**	96.5 [67–226]	105 [62–230]	102.5 [65–204]	105 [50–201]	83 [40–153]*	94.5 [59–153]*	109 [66–151]
Survivors	100 [68–226]	107.5 [73–230]	108.5 [66–204]	109.5 [56–201]	101.5 [40–159]* ^†^	94.5 [59–153]*	109 [66–151]
Non-Survivors	83 [33.5–206.5]	75.5 [26.5–195]	66 [21.5–192]*	105 [18–254]	28 [7–49] ^†^	-	-
**AT III (%)**	60±21	51±19*	48±20*	50±18*	54±22	68±24	70±26
Survivors	62±21	54±18*^†^	52±19*^†^	53±18*	56±21	68±24	70±26
Non-Survivors	54±23	38±19*^†^	32±19*^†^	40±16	35±28	-	-
**Albumin (g/L)**	24.4±2.3	23±2	23.7±2.2	24.9±1.8	25.1±2.3	26.6±1.7	26.8±1.5
Survivors	243±2.3	22.9±2.1	23.9±2.1	24.9±1.8	25.2±2.2	26.6±1.7	26.8±1.5
Non-Survivors	24.8±2.6	23.3±2.1	22.5±2.1	25±2.1	24.3±2.6	-	-
**PCT (ng/ml)**	5.6 [2–20.4]	4.7 [1.5–16.3]*	2.9 [1.4–17.8]*	2.9 [1.1–16.9]*	1.3 [0.4–2.6]*	1.6 [0.4–4.3]*	1.3 [0.4–2.6]*
Survivors	4.5 [2–20.6]	3.4 [1.4–16.3]*	2.7 [1.2–17.8]*	2.2 [0.9–17.2]*	2.2 [0.8–12.1]*	1.6 [0.4–4.3]*	1.3 [0.4–2.6]*
Non-Survivors	6.4 [3.7–7.2]	5.7 [4.3–13.6]	4 [2.4–14.3]	4.4 [1.7–14.4]	1.3 [1.1–4.3]	-	-
**PCR (mg/L)**	143 [65–237]	135 [50–231]	135 [45–229]	102 [52–217]	96 [49–166]*	55 [27–85]*	44 [20–89]*
Survivors	143 [45–248]	121 [42–237]	118 [40–236]	101 [34–237]	90 [31–157]*	55 [27–85]*	44 [20–89]*
Non-Survivors	143 [81–187]	152 [75–216]	152 [84–214	169 [62–217]	149 [100–193]	-	-
**TNFα (pg/ml)**	46.1 [21.2–113]			38.3 [11.4–52.8]*	32.5 [15.4–46.4]*	23.5 [15.2–35]*	
Survivors	76.6 [22.9–167]			39.9 [13.9–58.2]*	33.8 [20.9–49.7]*	23.5 [15.2–35]*	
Non-Survivors	24 [17.4–94			14.7 [10.5–48.8]*	15.4[10.4–32.5]*	-	
**IL-6 (pg/ml)**	228 [98.3–1091]			85.4 [61–171]*	99.1 [40–178]*	54.4 [29.7–141.4]*	
Survivors	233 [167–1450]			108.2 [69.3–406]*	108.1[68.8–193]*	54.4 [29.7–141.4]*	
Non-Survivors	98.3 [59.8–1091]			39.7 [38.3–165]	32.7[17–165]	-	
**IL-10 (pg/ml)**	28.1 [10.8–87.4]			20.1 [7.5–29.7]	12.2 [8.1–21]	10 [5.2–30.3]	
Survivors	39.5 [10.5–198.2]			21 [5.5–60.9]	11.8 [9.65–33.3]	10 [5.2–30.3]	
Non-Survivors	11.7 [11.5–28.1]			14.6 [10.7–20.1]	20.9[7.9–21]	-	

* Value statistically different to the baseline using statistical test for pairs data.

† Value statistically different between groups using statistical test for unpaired data.

## Discussion

It is well known that an adequate source control and an effective antibiotic treatment are the only etiological therapies able to improve patient survival [8,14,15]. However, in selected cases, extracorporeal blood purification therapies (e.g. HCO-CVVHD) may positively affect clinical outcome through multiple effects, including the reduction in inflammatory burden and restoring immunocompetence [16].

In this prospective, observational, cohort, multicenter study, a significant improvement in organ function as assessed by trends of SOFA score over time has been observed for patients with septic shock and AKI during treatment with HCO-CVVHD. The reduction in SOFA score was observed early during the treatment, persisting up to 48 hrs after discontinuation of HCO-CVVHD. Lactatemia and a higher KDIGO stage of AKI were independently associated to short-term mortality.

The effects of HCO membranes in septic patients with AKI have been tested previously, with conflicting results [17]. The lack of a standardized definition of HCO membranes (e.g. high permeability, super high-flux) and the different settings and/or treatment modalities may explain these non-homogeneous clinical results [7].

For standardization purpose, “high cut-off membranes” are nowadays defined as membranes with a cut-off value that approximates the molecular weight of albumin (i.e. 60 KDa) after blood exposure [12,13,18]. This feature is important to achieve clinically relevant clearance of “filterable” toxins, such as inflammatory mediators [7,19–21].

Several small experimental and clinical studies have shown that the application of extracorporeal therapies with HCO filters in septic patients with AKI may improve organ function. The pilot study by Morgera et al. showed significant reductions in APACHE II and Multiple Organ Dysfunction Score (MODS) in patients filtered with HCO membranes versus standard high-flux membranes [22]. In a retrospective study, we have previously shown a reduced need of vasoactive drugs/mechanical ventilation and better short term survival during HCO-CVVHD in patients with septic AKI sustained by gram-negative bacteria [23]. Furthermore, in a randomized controlled trial involving 30 septic patients with AKI, it was demonstrated that HCO-hemofiltration significantly reduced 48 hrs-SAPS II with respect to baseline [24]. Nevertheless, no definitive evidence of reduced long-term mortality has been established for HCO-CVVHD [6,7,23].

In our study, variations in SOFA score and reduced need for vasopressors were the hallmarks of efficacy of treatments in terms of organ dysfunction improvement. Indeed, we observed a significant SOFA score reduction (greater than 2.5 points) already at 48 hrs of HCO-CVVHD treatment (see Fig 2). Although the observational nature of this study does not allow to univocally advocate this effect to HCO-CVVHD, some considerations can be done. The observed reductions in SOFA score was greater than the mean reduction reported for similar septic patients not treated with HCO-CVVHD [10]. A more pronounced and earlier reduction in SOFA score has been observed among Survivors than non-Survivors patients (Fig 2). Of note, SOFA score lowered as earlier as in 6 hours in treated survivors, reaching significance in 12 hours: making it an early predictor of survival during HCO-CVHHD (see Fig 2). This observed reduction was more pronounced and earlier even to that described in literature for septic patients with AKI and a favorable ICU outcome not treated with HCO-CVVHD [10]. This may suggest a possible positive effect of HCO-CVVHD in our population.

Consistently, the improvement of SOFA score was associated with improved hemodynamics and a significant reduction in vasoactive need, in particular norepinephrine [23,25] (see Table 3). In Survivors, a significant reduction of norepinephrine dose was already evident 6 hours after HCO-CVVHD initiation. In several studies, hemodynamic stability has been correlated with the reduction of inflammatory mediators obtained with HCO [26]. In particular, an effective transmembrane removal of inflammatory mediators by means HCO, has been demonstrated for IL-6 and TNF-a [27–29]. Similarly, also in this cohort of septic patients, median values of TNF-a and IL-6 significantly lowered during HCO-CVVHD (p<0.05, see Table 3). We are aware that, because of the observational nature of this study, a causal correlation between the reduction of inflammatory mediators and HCO-CVVHD cannot be univocally defined. Interestingly, this effect was particularly evident for Survivors, paralleling the reduction in SOFA score and norepinephrine (Table 3). Indeed, considering that no differences were observed between groups for IL-6 or TNF-a levels before the HCO-CVVHD initiation, we cannot assume that baseline cytokine levels might be considered as outcome predictors in our patients. Nevertheless, the statistically significant reduction exclusively observed among survivors, might suggest that an early reduction of cytokine levels during the treatment might be a potential predictor for positive outcome during HCO-CVVHD. Although clinical fascinating, this cytokines reduction during extracorporeal treatments has not been univocally related to positive outcome in septic patients; furthermore, considering the high costs and the limited laboratory feasibility for cytokine analysis, the reduction in circulating cytokines might unlikely be proposed as predictor in the ICU clinical routine practice.

The observed ICU-mortality was lower to that expected from literature and based to baseline APACHE II score. In particular, with a baseline APACHE II score of 37.55±3.55 (37.6±3.06 and 38±2.92 for Survivors and non-Survivors, respectively), a 71% mortality rate could be expected in this population. Interestingly, overall, we observed an in-hospital mortality rate of 39.5%. The identification of specific baseline patients’ characteristics associated with early (72 hrs) ICU-mortality during HCO-CVVHD may be of prognostic values for physicians using this invasive extracorporeal treatment. The initial KDIGO-AKI stage and serum lactate levels were independently associated with survival at 72 hour of HCO-CVVHD. The baseline values of serum lactate concentration were different between the groups: 2.4 vs 6.3 mEq/l in Survivors and non-Survivors, respectively. In light of this, it would be expected that serum lactates concentration (particularly values higher than 5.2 mmol/l) and AKI stage correlate with mortality. It can be thus speculated that early initiation of extracorporeal treatment, at lower degrees of global hypoxia and organ dysfunction, may positively affect the outcome [30–33]. In light of this, even though the observational nature of the study does not allow to correlate clinical improvements with HCO-CVVHD, our results suggest that HCO-CVVHD may retain a positive clinical effect in patients with septic AKI. Clearly, results on these outcomes cannot be generalized for all septic patients. Indeed, although the mean SOFA score observed by Carbonell et al.[10] (used for sample size calculation) is consistent with those observed in other important studies on similar population as ALBIOS [34], EUPHAS [35]or even in the most recent ABDOMIX [36], a wide dispersion of SOFA score values has been observed, also within each single study. As a consequence, SOFA score utilized for sample size evaluation might not necessarily align that observed in our septic shock population, and thus potentially under-powering this study.

## Conclusions

HCO-CVVHD was associated with a significant reduction in SOFA score in a cohort of patients with septic shock and AKI. This effect was evident early during the treatment and associated with a significant reduction of circulating inflammatory mediators, such as IL-6 and TNF-a. Serum lactate levels and the initial stage of AKI were statistically associated to 72 hrs-ICU mortality.

## Supporting information

S1 Table Complete data set. All clinical data for each patients are reported in this table.
(PDF)
