# Supplementary material for: Organ dysfunction during continuous veno-venous high cut-off hemodialysis in patients with septic acute kidney injury: A prospective observational study
Source: PLoS One. 2017 Feb 16;12(2):e0172039. doi: 10.1371/journal.pone.0172039 (PMC5313216; doi:10.1371/journal.pone.0172039)
Supplement: S1 Table — All clinical data for each patients are reported in this table. (PDF) [file pone.0172039.s001.pdf]

A	et	uomo	ORIGINERE	ORIGINEAD	ORIGINEUF	ORIGINEEM	ORIGINESN	ORIGINEAlt
paz 2	67	0	1	0	0	0	0	0
paz 3	78	1	1	0	0	0	0	0
paz 7	46	0	0	1	0	0	0	0
paz 8	82	1	0	1	0	0	0	0
paz 11	79	1	1	0	0	0	0	0
paz 12	80	1	1	0	0	0	0	0
paz 13	78	0	0	0	0	1	0	0
paz 14	78	1	0	1	0	0	0	0
paz 26	87	1	0	1	0	0	0	0
paz 27	83	0	0	0	1	0	0	0
paz 29	64	1	0	0	0	1	0	0
paz 31	60	1	0	1	0	0	0	0
paz 32	70	1	1	0	0	0	0	0
paz 33	75	1	1	0	0	0	0	0
paz 34	86	1	0	0	0	1	0	0
paz 35	86	0	0	0	1	0	0	0
paz 36	84	0	0	0	1	0	0	0
paz 4	74	0	0	0	0	0	0	1
paz 5	50	1	1	0	0	0	0	0
paz 6	67	1	0	0	0	1	0	0
paz 9	63	1	0	1	0	0	0	0
paz 10	72	1	0	1	0	0	0	0
paz 15	75	1	1	0	0	0	0	0
paz 16	61	1	1	0	0	0	0	0
paz 17	81	1	0	0	0	1	0	0
paz 18	70	1	0	0	0	1	0	0
paz 19	75	1	0	0	1	0	0	1
paz 20	77	0	0	0	0	0	0	0
paz 21	79	1	0	0	0	0	0	1
paz 22	70	0	1	0	0	0	0	0
paz 23	75	1	0	1	0	0	0	0
paz 24	82	0	1	0	0	0	0	0
paz 25	63	1	0	1	0	0	0	0
paz 28	72	0	0	0	0	1	0	0
paz 30	38	1	0	0	0	0	0	1
paz 37	64	1	0	0	0	0	0	1
paz 38	70	1	0	0	0	0	0	1
paz 39	46	1	1	0	0	0	0	0

itcontrollat	itnoncontro	BPCOLieve	BPCOMode	BPCOGrave	diabetecon	diabetenor	IRADialisi	IRANodialis
0	0	0	1	0	0	0	0	0
0	1	0	0	0	0	0	0	0
0	0	0	0	0	0	0	0	0
1	0	0	0	0	0	0	0	1
1	0	1	0	0	0	0	1	0
1	0	1	0	0	0	0	0	0
0	0	0	0	0	0	0	1	0
0	0	0	1	0	0	0	0	1
1	0	0	0	0	0	0	0	0
1	0	0	0	0	1	0	0	0
1	0	1	0	0	0	0	0	0
1	0	0	0	0	1	0	0	0
1	0	0	1	0	1	0	0	0
0	0	0	0	0	0	0	0	0
1	0	0	0	0	0	0	0	1
1	0	0	0	0	0	0	0	0
1	0	0	0	0	1	0	0	0
1	0	0	0	0	0	0	0	0
1	0	0	0	0	0	1	0	1
1	0	0	0	0	0	1	0	0
1	0	0	0	0	0	0	0	0
1	0	0	0	0	1	0	0	0
0	1	0	0	0	0	0	0	1
0	1	1	0	0	0	1	1	0
1	0	0	1	0	0	0	0	0
1	0	1	0	0	1	0	0	1
1	0	0	0	0	0	0	0	1
0	0	0	1	0	1	0	0	1
0	1	1	0	0	0	0	0	1
1	0	0	0	0	0	0	0	0
1	0	1	0	0	0	0	0	0
1	0	0	0	0	0	0	0	0
0	1	1	0	0	0	0	0	0
1	0	1	0	0	1	0	0	0
0	0	0	0	0	0	0	0	0
0	0	0	0	0	0	0	0	0
1	0	0	0	0	0	0	0	1
1	0	1	0	0	0	0	0	0

NYHAIII	NYHAIIIIV	Deficitsisto	Deficitdiast	Coronarie	Perferica	SNC	Metastasi	Nometasta
0	0	0	0	0	0	0	0	0
0	0	0	0	0	1	0	0	0
0	0	0	0	0	0	0	0	0
0	0	0	0	1	0	0	0	1
0	1	0	0	1	1	1	0	0
0	1	0	0	1	1	1	0	0
0	0	0	0	1	0	0	0	0
0	1	0	0	1	0	0	0	0
0	0	0	0	0	0	0	0	0
0	0	0	0	0	0	0	0	1
1	0	0	0	1	0	0	0	0
0	1	1	0	1	0	0	0	0
1	0	1	0	0	0	0	0	0
0	0	0	0	0	0	0	0	0
1	0	1	0	0	0	0	0	0
0	0	0	0	0	0	0	0	0
0	0	0	0	0	0	0	0	1
0	0	0	0	0	0	0	0	0
0	0	0	0	0	0	1	0	0
0	0	0	0	0	0	0	0	0
1	0	0	0	1	0	0	0	0
0	0	0	0	0	0	0	0	0
0	1	0	0	1	0	0	0	0
1	0	0	0	1	1	0	0	0
0	0	0	0	0	0	0	0	0
0	0	0	0	0	0	0	0	0
0	0	0	0	1	0	0	0	0
1	0	0	0	0	0	0	0	0
0	0	0	0	0	1	0	0	0
0	0	0	0	1	0	0	0	0
0	1	0	0	1	1	0	0	0
0	0	0	0	1	0	0	0	0
0	0	0	0	0	0	0	0	0
0	1	1	0	1	0	0	0	0
0	0	0	0	0	0	0	0	0
0	1	1	0	1	0	0	0	0
0	1	1	0	0	0	0	0	0
1	0	0	0	0	0	0	0	0

Ematogene	Immunode	Disturboco	KDIGOT0	APACHEII	decesso72l	SOFATOTt0	SOFATOTt6	SOFATOTt1
0	0	0	2	19	0	12	8	8
0	0	0	2	20	0	13	13	12
0	0	0	2	16	0	16	14	9
0	0	0	3	30	0	12	11	10
0	0	0	3	22	1	13	13	15
0	0	0	3	22	0	15	13	10
0	0	0	3	13	0	9	7	7
0	0	1	3	20	0	16	15	13
0	0	0	3	36	0	8	10	9
0	0	0	2	19	1	10	7	8
0	0	0	3	31	0	15	13	13
0	0	0	2	20	0	10	9	7
1	0	0	2	40	0	16	15	12
0	0	0	1	30	0	12	12	10
0	0	0	3	20	0	12	11	9
0	0	0	1	25	0	13	13	10
0	0	0	1	26	0	13	12	10
0	0	0	2	22	0	10	9	9
0	0	0	3	21	0	18	14	14
0	0	0	3	12	1	10	10	10
0	0	0	2	19	0	8	7	5
0	0	0	3	29	1	16	16	16
0	1	0	3	26	0	15	13	11
0	0	0	3	19	1	14	14	12
0	0	0	2	17	0	14	14	13
0	0	0	3	10	0	6	5	3
0	0	0	3	37	1	15	17	16
0	0	0	1	18	0	8	10	8
0	0	0	3	21	0	15	12	12
0	0	0	3	24	1	6	6	6
0	0	0	3	25	0	14	12	13
0	0	0	2	24	0	11	7	6
0	0	0	1	16	0	7	8	6
0	0	0	3	24	1	10	10	10
0	0	0	2	24	0	13	11	11
0	0	0	2	35	0	12	11	11
0	0	0	2	31	0	8	9	9
0	0	0	1	31	0	6	5	5

SOFATOTt2	SOFATOTt4	SOFATOTtp	SOFATOTtp	SOFAcardic	SOFAcardic	SOFAcardic	SOFAcardic	SOFAcardic
8	6	3	3	4	4	4	3	3
10	8	7	5	4	4	4	4	4
7	4	1	1	4	3	1	1	0
7	6	4	4	4	4	3	2	1
17	18			2	2	2	4	4
10	9	6	7	4	4	3	3	3
7	7	7	7	3	3	3	3	3
10	9	6	5	4	4	4	3	3
5	5	10	10	4	4	4	1	
8	9			4	4	4	4	4
13	15			4	4	4	4	4
7	7	7	7	2	1	0	0	0
11	8	6	5	3	3	3	3	3
10	10	9	8	3	3	3	3	3
9	8	7	7	3	3	3	3	3
8	6	7	8	4	4	3	0	0
6	6	7	8	4	4	4	2	1
7	6	5	6	4	4	4	3	3
15	9	9	9	4	4	4	4	3
10	11			4	4	4	4	4
4	2	1	3	4	4	3	2	1
16				4	4	4	4	
11	8	7	7	4	4	3	3	3
9	9			4	4	4	4	3
13	15			4	4	4	4	4
4		3	3	0	0	0	0	
				4	4	3		
9		5	4	2	2	2	2	
11	9	13	17	4	4	4	4	4
6		7	5	2	2	2	2	
13	9	8	8	5	3	3	3	1
		7	5	4	1	0		
6		11	10	3	3	3	3	
15	14			1	1	1	4	4
12	13	9	9	4	4	4	4	4
11	11	7	7	4	4	4	4	4
7	7	8	8	2	2	2	2	2
4	4	4	5	0	0	0	0	0

SOFAcardic	SOFAcardic	SOFArenet	SOFArenet	SOFArenet	SOFArenet	SOFArenet	SOFArenet	SOFArenet
3	3	0	0	0	0	0	0	0
4	3	1	1	1	2	1	1	1
0	0	2	1	1	0	0	0	0
0	0	2	1	1	0	0	0	0
		1	0	0	0	0		
3	3	1	1	0	0	0	1	2
2	2	0	0	0	0	0	0	0
3	3	0	0	0	0	0	0	0
2	2	2	1	1	0		0	0
		1	0	0	0	0		
		1	1	1	1	1		
1	0	0	0	0	0	0	0	0
0	0	4	4	3	1	1	2	2
3	1	0	0	0	0	0	0	0
3	2	2	1	0	0	0	0	0
0	1	3	2	2	1	0	1	2
1	1	2	1	0	0	0	1	1
3	3	3	3	3	2	1	1	1
3	3	3	2	2	3	2	2	2
		1	1	1	1	1		
0	0	2	2	2	2	1	1	2
		1	1	1	1			
2	2	2	1	1	0	1	1	1
3	3	1	1	1	0	0	0	0
		1	1	1	0	0		
0	0	2	1	1	1		0	0
		0	0	0				
0	0	0	0	0	1		1	0
4	4	3	2	2	2	1	0	0
2	2	1	1	1	1		2	1
1	1	2	2	1	2	1	0	0
0	0	1	0	1			1	1
3	3	0	1	0	0		2	2
		1	0	0	0	0		
0	0	1	2	1	1	1	2	2
3	3	1	1	0	0	0	1	1
2	2	4	4	3	3	3	4	4
0	0	1	1	1	1	1	1	1

SOFAneuro	SOFAneuro	SOFAneuro	SOFAneuro	SOFAneuro	SOFAneuro	SOFAneuro	SOFArespt(	SOFArespt6
0	0	0	0	0	0	0	4	2
2	2	2	2	1	0	0	3	3
3	2	2	1	1	0	0	3	3
2	2	2	1	1	0	0	4	4
3	3	3	4	4			2	2
3	2	2	2	1	0	0	3	2
1	1	1	1	1	1	1	3	1
3	2	2	1	1	0	0	3	3
1	3	3	3		4	4	0	2
3	2	2	1	1			2	1
2	1	1	1	1			2	2
2	2	1	2	2	1	1	4	4
4	4	3	3	1	0	0	4	3
4	4	4	4	4	4	4	3	3
3	3	4	4	3	1	2	3	3
1	2	2	2	0	1	0	3	2
3	3	4	2	1	1	2	4	3
2	1	1	1	1	0	1	1	1
3	3	3	3	1	1	1	4	3
1	1	1	1	2			1	1
1	0	0	0	0	0	0	1	1
3	3	3	3				2	2
3	2	1	1	1	0	0	3	2
3	3	2	1	1	1	1	3	3
3	3	3	3	3			2	2
3	3	2	2		2	2	1	1
3	3	3					4	4
3	3	2	2		1	1	0	2
2	2	2	2	2	2	3	4	4
0	0	0	0		0	0	3	3
2	2	2	2	2	2	2	2	2
1	1	1			1	1	3	2
1	1	1	1		2	1	1	1
1	1	1	2	2			3	3
4	2	2	2	2	0	0	0	0
4	4	4	4	4	0	0	2	1
1	1	1	0	0	0	0	0	1
1	1	1	1	1	1	1	4	3

SOFArespt1	SOFArespt2	SOFArespt4	SOFAresptp	SOFAresptp	SOFAepato	SOFAepato	SOFAepato	SOFAepato
1	1	0	0	0	2	1	2	2
3	2	1	1	1	2	2	2	1
1	1	0	0	0	2	2	2	1
4	4	3	2	2	0	0	0	0
4	3	4			2	2	2	2
2	2	2	1	1	1	1	0	0
1	1	1	1	1	0	0	0	0
1	1	0	0	0	2	2	2	1
1	1		2	2	1	0	0	0
2	2	2			0	0	0	1
2	2	2			3	3	3	3
4	3	3	2	2	2	2	2	2
3	3	3	3	3	1	1	1	1
3	3	3	2	2	0	0	0	0
3	1	1	1	1	0	0	0	1
3	3	3	2	3	0	1	0	0
4	2	4	3	3	0	1	1	0
1	1	1	1	1	0	0	0	0
3	3	2	2	2	2	1	1	1
1	1	1			1	1	1	1
0	0	0	0	0	0	0	0	0
2	2				3	3	3	3
2	2	2	1	1	2	1	1	1
3	2	2	1	1	2	2	1	1
2	2	2			2	2	1	1
0	0		0	0	0	0	0	0
4					2	3	3	
1	1		1	2	1	1	1	1
4	3	2	2	3	1	0	0	0
3	3		1	1	0	0	0	0
2	2	2	2	2	1	1	1	2
2			2	1	0	0	0	
0	0		1	1	0	0	0	1
3	4	3			0	1	1	1
0	0	0	2	2	2	2	2	2
1	1	1	1	2	1	1	2	2
2	1	1	1	1	0	0	0	0
3	2	3	2	3	0	0	0	0

SOFAepato	SOFAepato	SOFAepato	SOFAcoagt	SOFAcoagt	SOFAcoagt	SOFAcoagt	SOFAcoagt	SOFAcoagt
1	0	0	3	3	3	3	3	3
1	1	0	0	0	0	0	0	0
1	0	0	2	2	2	2	3	2
0	0	0	0	0	0	0	1	2
2			3	4	4	4	4	
0	0	0	3	3	3	3	3	1
0	0	0	2	2	2	2	2	3
1	0	0	4	4	4	4	4	3
	2	2	0	0	0	0		0
1			0	0	0	0	1	
3			3	2	2	2	4	
2	1	1	0	0	0	0	0	0
0	0	0	0	0	0	0	0	1
0	0	0	2	2	2	2	2	2
0	0	0	1	1	1	1	1	2
0	1	0	2	2	2	2	2	2
0	0	0	0	0	0	0	0	1
0	0	0	3	3	3	3	3	3
0	0	0	2	1	1	1	1	1
1			2	2	2	2	2	
0	0	0	0	0	0	0	0	0
			3	3	3	3		
1	1	1	0	0	0	0	1	1
1	1	0	1	1	1	1	2	2
2			2	2	2	3	4	
	0	0	0	0	0	1		1
			2	3	3			
	1	0	2	2	2	2		1
0	2	3	1	0	0	0	0	3
	2	1	0	0	0	0		0
1	1	1	2	2	2	2	2	2
	0	0	2	3	2			3
	1	1	2	2	2	2		2
1			4	4	4	4	4	
3	2	2	2	1	2	3	3	3
2	2	1	0	0	0	0	0	0
0	0	0	1	1	1	1	1	1
0	0	0	0	0	0	0	0	0

SOFAcoagt	NorAdrt0	NorAdrt6	NorAdrt12	NorAdrt24	NorAdrt48	NorAdrtpo	NorAdrtpo	Adret0
3	.4	.3	.2	.1	.05	.05	0	.1
0	.4	.4	.3	.3	.2	.2	.15	.1
1	.25	.1	0	0	0	0	0	.13
2	.3	.25	.2	.05	0	0	0	0
	.04	.03	.07	.2	.2			.03
1	.2	.2	.2	.1	.1	.1	.1	.1
3	.2	.2	.2	.15	.1	.1	.1	.1
2	.3	.3	.28	.25	.1	.04	.02	0
0	.25	.25	.25	.32		.25	.35	0
	.5	.3	.2	.2	.2			0
	.25	.25	.25	.03	.1			0
0	.2	.1	0	0	0	0	0	0
0	.1	.1	.1	.04	.04	0	0	0
2	.1	.1	.1	.1	.1	.06	0	0
2	.1	0	0	0	.06	.06	0	0
2	.15	.1	.1	0	0	0	0	0
1	.15	.1	.15	0	0	0	0	0
3	.4	.3	.3	.1	.1	.05	.05	0
1	.2	.2	.2	.2	.2	.2	.2	0
	.1	.2	.3	.3	.3			0
1	.3	.3	.2	.05	0	0	0	0
	.4	.35	.35	.35				0
1	.3	.3	.2	.1	.05	.05	.05	.1
2	.25	.2	.2	.2	.2			.15
	.3	.3	.3	.3	.3			.05
1	.1	0	0	0	0	0	0	0
	.2	.2	.1					.08
1	.1	0	0	0		0	0	0
4	.25	.3	.3	.3	.3	.3	.3	0
0	.15	.2	.2	.2				0
2	.25	.1	0	0	0	0	0	0
2	.15	0	0			0	0	0
2	.08	.08	.08	.08		.08	0	0
	.15	.05	.05	.25	.2			.03
3	.6	.6	.6	.6	.6	.2	.15	0
0	.35	.15	.15	.15	.15	.1	.1	.18
1	.1	0	0	0	0	0	0	0
0	.1	0	0	0	0	0	0	0

Adret6	Adret12	Adret24	Adret48	Adretpost2	Adretpost4	Dobutt0	Dobutt6	Dobutt12
.1	.1	0	0	0	0	2.5	3	3
.1	.1	0	0	0	0	0	0	0
.05	0	0	0	0	0	0	0	0
0	0	0	0	0	0	0	0	0
.03	.03	.05	.2			3	3	3
.1	.05	0	0	0	0	3	3	3
.05	0	0	0	0	0	3	3	3
0	0	0	0	0	0	3	3	3
0	0	0		0	0	0	0	0
0	0	0				0	0	0
0	0	.05	0			0	0	0
0	0	0	0	0	0	0	0	0
0	0	0	0	0	0	0	0	0
0	0	0	0	0	0	0	0	0
0	0	0	0	0	0	0	0	0
0	0	0	0	0	0	0	0	0
0	0	0	0	0	0	0	0	0
0	0	0	0	0	0	0	0	0
0	0	0	0	0	0	0	0	0
.1	.1	.1	.1			0	0	0
0	0	0	0	0	0	0	0	0
0	0	0				0	0	0
.1	0	0	0	0	0	3	3	3
.1	.1	.1	.1			3	3	3
.1	.12	.12	.1			0	0	0
0	0	0	0	0	0	0	0	0
.08	.1					0	0	0
0	0	0		0	0	0	0	0
0	0	0	0	0	0	0	0	0
0	0	0				0	0	0
0	0	0	0	0	0	0	0	0
0	0			0	0	0	0	0
0	0	0	0	0	0	0	0	0
		.2	.03			0	0	0
0	0	0	0	0	0	0	0	0
0	0	0	0	0	0	0	0	0
0	0	0	0	0	0	0	0	0
0	0	0	0	0	0	0	0	0

Dobutt24	Dobutt48	Dobuttpos	Dobuttpos	Dopat0	Dopat6	Dopat12	Dopat24	Dopat48
3	3	3	3	3	3	3	3	3
0	0	0	0	0	0	0	0	0
0	0	0	0	0	0	0	0	0
0	0	0	0	0	0	0	0	0
3	3			0	0	0	0	0
3	3	3	3	0	0	0	0	0
3	3	3	3	0	0	0	0	0
3	4	2	2	5	5	5	5	5
0		0	0	0	0	0	0	
0				0	0	0	0	
24	0			0	0	0	0	0
0	0	0	0	0	0	0	0	0
0	0	0	0	0	0	0	0	0
0	0	0	0	0	0	0	0	0
0	0	0	0	10	10	10	10	0
0	0	0	0	0	0	0	0	0
0	0	0	0	10	10	10	5	0
0	0	0	0	3.5	3	3	3	3
0	0	0	0	0	0	0	0	0
0	0			3	3	3	3	3
0	0	0	0	0	0	0	0	0
0				0	0	0	0	
3	3	3	3	0	0	0	0	0
3	3	3	3	0	0	0	0	0
0	0			0	0	0	0	0
0	0	0	0	3	3	3	0	
				0	0	0		
0		0	0	2	4	2	2	
0	0	0	0	0	0	0	0	0
0		0	0	2.5	2.5	2.5	2.5	
0	0	0	0	0	0	0	0	0
		0	0	0	0	0		
0	0	0	0	0	0	0	0	0
0	0			0	0	0	0	0
0	0	0	0	0	0	0	0	0
0	0	0	0	.5	.5	.5	.5	.5
0	0	0	0	4	0	0	0	0
0	0	0	0	0	0	0	0	0

Dopatpost	Dopatpost	Enoximt0	Enoximt6	Enoximt12	Enoximt24	Enoximt48	Enoximtpo	Enoximtpo
3	3	0	0	0	0	0	0	0
0	0	0	0	0	0	0	0	0
0	0	0	0	0	0	0	0	0
0	0	0	0	0	0	0	0	0
		0	0	0	0	0		
0	0	2	2	2	2	2	2	2
0	0	0	0	0	0	0	0	0
5	4	0	0	0	0	0	0	0
0	0	0	0	0	0		0	0
		0	0	0	0			
		1	1	1	1	0		
0	0	0	0	0	0	0	0	0
0	0	0	0	0	0	0	0	0
0	0	0	0	0	0	0	0	0
0	0	0	0	0	0	0	0	0
0	0	0	0	0	0	0	0	0
0	0	0	0	0	0	0	0	0
3	3	0	0	0	0	0	0	0
0	0	0	0	0	0	0	0	0
		0	0	0	0	0		
0	0	0	0	0	0	0	0	0
		0	0	0	0			
0	0	0	4	4	3	3	3	3
0	0	3	3	3	3	3	3	3
		5	3	3	3	0		
0	0	0	0	0	0	0	0	0
		0	0	0				
0	0	0	0	0	0		0	0
0	0	0	0	0	0	0	0	0
2.5	2.5	0	0	0	0		0	0
0	0	0	0	0	0	0	0	0
0	0	3	3	3			0	0
0	0	0	0	0	0	0	0	0
		0	0	0	0	0		
0	0	1.5	0	0	0	0	0	0
.5	.5	2.5	0	0	0	0	0	0
0	0	0	0	0	0	0	0	0
0	0	0	0	0	0	0	0	0

AVPt0	AVPt6	AVPt12	AVPt24	AVPt48	AVPtpost2	AVPtpost4	PAST0	PAST6
0	0	0	0	0	0	0	77	90
0	0	0	0	0	0	0	95	100
0	0	0	0	0	0	0	96	101
0	0	0	0	0	0	0	87	102
4	0	4	3	3			65	120
0	0	0	0	0	0	0	70	80
2	1	0	0	0	0	0	100	100
0	0	0	0	0	0	0	100	90
0	0	0	0		0	0	105	107
1	.5	.3	0	0			148	108
70	90	85	65	80			60	80
0	0	0	0	0	0	0	135	143
0	0	0	0	0	0	0	155	127
0	0	0	0	0	0	0	148	118
0	0	0	0	0	0	0	140	136
0	0	0	0	0	0	0	140	120
0	0	0	0	0	0	0	120	117
0	0	0	0	0	0	0	95	134
0	0	0	0	0	0	0	110	110
0	0	0	0	0			95	110
0	0	0	0	0	0	0	121	120
0	0	0	0				109	74
0	0	0	0	0	0	0	130	145
3	3	3	3	3	3	3	70	85
2	2	2	2	3			80	110
0	0	0	0	0	0	0	100	115
3	3	4					70	68
0	0	0	0		0	0	115	115
0	0	0	0	0	0	0	115	105
0	0	0	0		0	0	100	95
0	0	0	0	0	0	0	80	100
0	0	0			0	0	115	110
0	0	0	0	0	0	0	80	90
0	0	0	0	0			100	70
0	0	0	0	0	0	0	60	80
0	0	0	0	0	0	0	75	95
0	0	0	0	0	0	0	140	140
0	0	0	0	0	0	0	100	120

PAST12	PAST24	PAST48	PASTpost2	PASTpost4	PADT0	PADT6	PADT12	PADT24
97	120	150	140	135	40	50	45	50
110	110	120	160	120	30	50	40	50
105	92	112	100	130	45	56	57	45
101	111	135	111	110	45	53	51	50
110	92	50			35	35	40	30
110	110	100	100	100	35	30	40	50
105	95	88	100	100	43	45	45	45
90	90	90	110	90	40	40	40	50
92	97	90	85	83	55	54	56	47
100	100	100			53	58	60	90
110	100	100	130	140	46	45	65	50
145	146	125	102	105	60	66	62	65
179	112	150	110	160	87	87	84	52
128	121	119	159	131	63	53	57	54
110	114	150	160	155	50	42	40	40
130	140	189	169	130	60	65	65	60
170	102	150	144	125	56	56	50	50
110	120	150	130	160	35	50	45	43
133	120	120	150	145	60	50	63	54
115	80	110			50	60	65	40
150	130	130	120	130	50	60	70	65
84	0				50	38	37	57
113	120	115	117	113	60	40	45	50
75	100	100			30	30	54	40
80	90	90	110	110	45	60	50	40
118	166	140	130	130	56	70	61	88
70					35	38	40	
105	90	110	100	105	69	58	45	40
85	100	100	115	135	70	40	40	45
90	102				60	60	57	63
120	120	100	100	100	45	50	60	55
110	110	120	130	110	35	30	40	
80	110	120	150	90	50	50	50	60
80	80	70			30	30	40	30
110	100	100	130	140	33	50	60	40
120	100	100	100	95	45	60	60	50
120	120	150	135	150	80	50	50	50
100	100	120	90	110	60	70	70	60

PADT48	PADTpost2	PADTpost4	PAMT0	PAMT6	PAMT12	PAMT24	PAMT48	PAMTpost2
77	60	55	51	55	63	75	110	90
85	65	90	50	55	60	65	65	75
60	50	60	60	67	72	58	75	61
65	52	50	67	69	68	68	70	62
30			45	50	60	38	31	
45	45	45	50	50	65	70	75	75
35	45	45	58	65	67	70	50	50
45	55	45	70	70	70	73	75	75
	46	50	72	72	57	62	61	60
90			81	76	72	90	90	
50			60	80	70	95	90	95
58	41	56	70	91	79	84	76	63
65	60	70	106	103	107	63	85	70
55	60	48	85	71	77	79	77	89
51	61	70	83	80	60	67	69	70
60	72	60	84	81	87	87	111	99
54	60	50	74	70	73	64	75	81
55	60	60	55	71	62	61	75	75
55	55	55	80	70	81	72	70	65
40			67	70	70	60	70	
65	60	80	69	80	90	75	75	80
			63	46	48	73		
45	54	50	75	60	65	65	65	65
40			50	50	51	55	55	
50			56	80	65	57	63	65
	70	90	69	85	80	114	90	80
			43	45	50			
	55	50	55	73	63	60	65	70
45	40	45	65	60	55	54	55	60
			75	70	70	72		
55	60	50	45	66	80	76	70	73
	40	20	62	57	63	70	70	70
	60	50	60	63	60	76	80	90
30			53	43	53	47	43	
50	60	55	41	55	60	55	60	75
50	45	45	55	70	80	66	66	75
60	50	60	95	80	80	80	85	78
80	50	70	73	83	80	73	93	63

PAMTpost4	FCT0	FCT6	FCT12	FCT24	FCT48	FCTpost24	FCTpost48	PVCT0
89	135	125	123	100	145	102	96	2
60	100	75	95	90	85	65	90	10
75	113	97	76	98	91	100	90	10
72	80	78	76	79	85	72	84	13
	80	80	80	91	90			14
75	85	85	85	85	85	85	85	10
50	90	90	89	80	90	90	90	18
60	80	80	80	80	80	75	75	17
60	81	88	97	85	88	93	95	12
	140	140	140	147	147			10
95	82	80	75	70	75	75	75	9
71	80	78	76	69	78	88	87	10
90	90	93	88	74	73	80	80	10
70	65	62	69	69	69	63	56	10
70	91	92	75	79	69	70	70	9
64	66	88	64	80	79	61	63	10
78	76	72	70	70	69	93	68	15
90	70	70	77	75	74	70	75	14
60	80	90	80	88	75	55	70	12
	96	98	100	57	90			19
82	100	115	94	85	85	80	64	6
	111	105	111	107				12
65	95	85	89	80	80	80	80	9
	80	80	90	90	90			10
65	80	80	80	80	70	65	65	18
103	99	95	110	75	76	100	80	12
	80	80	80					14
70	95	105	95	75	75	85	80	12
70	104	100	95	110	103	65	90	17
	100	84	98	102				4
66	75	80	80	80	80	85	70	14
50	70	60	70	70	70	70	70	10
63	80	95	70	70	70	105	110	10
	70	50	60	75	60			20
80	105	90	110	100	90	105	95	12
70	80	75	80	85	75	80	90	6
90	84	96	110	66	62	64	65	10
83	120	90	100	80	70	80	80	14

PVCT6	PVCT12	PVCT24	PVCT48	PVCTpost2	PVCTpost4	FRT0	FRT6	FRT12
10	8	7	11	6	6	20	24	10
13	7	7	8	12	12	20	20	15
10	9	10	10	12	10	20	15	12
13	13	12	12	10	10	26	9	8
16	15	19	14			16	16	15
7	10	20	14	14	14	33	18	15
17	16	14	11	9	8	14	15	15
21	22	20	23	13	13	23	23	14
8	8	12	12	8	14	12	12	12
13	7	17	17			22	20	18
13	13	14	18	15	15	19	16	15
11	15	14	14	13	13	18	14	19
11	11	11	14	11	12	15	15	14
11	11	9	9	10	10	11	10	12
7	6	6	7	7	7	15	17	11
11	11	9	9	10	10	21	31	19
16	17	20	21	14	14	12	12	12
12	9	10	10	10	12	16	20	25
12	12	10	10	10	10	17	16	16
16	16	12	10			13	19	19
14	12	11	11	11	11	22	21	27
12	12	12				22	16	16
9	6	7	8	8	8	11	14	16
12	12	18	16			19	19	22
22	18	20	18	18	18	12	14	14
14	12	12	12	13	14	17	18	14
14	15					10	12	19
14	12	12	12	13	14	20	20	12
10	9	12	6	12	11	13	18	18
12	10	11				25	25	23
14	11	17	14	12	13	23	20	17
10	10	11	11	12	8	24	22	23
15	9	11	13	15	10	12	12	12
15	10	10	12			14	14	14
14	15	14	13	10	5	10	10	10
9	8	15	13	7	7	14	18	10
11	15	14	14	13	13	18	19	19
12	9	10	10	10	12	30	22	23

FRT24	FRT48	FRTpost24	FRTpost48	SatO2T0	SatO2T6	SatO2T12	SatO2T24	SatO2T48
8	31	27	28	97.8	98.5	100	98.7	100
20	16	23	24	100	98	100	100	100
15	15	23	22	99	100	100	97	98
8	12	15	16	97.5	97	97	98	99
15	15			98	93	100	100	85
12	12	11	12	97	100	100	100	100
15	20	21	21	100	100	99.9	97	100
14	25	21	20	99.7	99	100	99.1	99
12	11	11	16	98	99	99	98.7	99
23	23			94	98	98	100	100
15	14	14	14	98	99	99	98	98
15	15	15	15	98	96	97	100	98
15	17	20	23	97	98	98	96	95
12	12	12	12	99	99	99	99	99
12	16	15	12	100	98	100	100	100
20	25	22	20	98	99	97	97	95
12	12	12	10	93	100	100	100	100
27	19	16	25	99	100	100	98	100
16	23	22	20	97	100	100	98	97
17	18			98.5	100	100	98	98.6
25	24	18	19	99	99	100	100	100
16				100	100	100	100	
15	14	14	14	99	97	100	100	100
18	21			100	99	99	99	98
10	10	11	11	96	98	98	96	97
14	15	18	20	99	100	99	100	100
				92	100	98		
16		14	13	100	100	100	100	100
18	18	15	18	98	100	99	98	95.3
28				98	98	98	98	
23	20	20	17	98	98	99	98	98
		24	21	99.2	100	99.2	100	100
15		10	15	98.6	98	97	99	100
15	14			95	97	95	91.5	97.7
20	10	22	28	98	99.1	98.5	98.7	98.7
18	19	15	18	97	97	100	99	99
20	21	19	15	96	99	98	98	98
18	26	26	20	100	100	100	100	100

SatO2Tpos	SatO2Tpos	PaO2FiO2T	PaO2FiO2T	PaO2FiO2T	PaO2FiO2T	PaO2FiO2T	PaO2FiO2T	PaO2FiO2T
100	100	100	248	360	377	738	668	516
100	100	200	287	287	288	245	255	274
97	100	160	234	234	574	238	297	350
98	98	170	240	210	190	300	300	300
		100	75	100	68	78		
100	100	120	240	240	235	300	300	300
100	100	244	250	272	215	295	300	300
97.9	98.8	302	415	367	365	382	332	260
95	100	400	293	385	353	360	261	231
		165	330	240	240	240		
100	100	210	287	227	265	222	300	300
100	99	90	67	78	173	152	265	218
95	94	80	188	184	186	121	180	176
100	100	159	145	149	151	150	232	200
100	100	200	200	193	350	400	344	350
100	100	164	205	175	125	140	240	110
100	100	100	173	100	227	83.9	103	140
100	98	400	423	416	383	377	430	436
97	97	97	121	108	120	130	335	330
		322	440	445	350	290		
100	100	335	370	320	350	350	410	417
		576	300	300	300			
100	100	240	300	300	250	300	300	300
		350	370	300	350	320		
100	100	210	201	224	236	252	300	300
100	100	440	300	321	300	350	560	580
		80	80	90				
100	100	475	287	396	300	350	360	420
98	96	99.6	75	76	110	129	277	197
		155	196	240	163			
99	100	260	240	277	297	203	199	240
100	98.6	198.4	256	258	260	265	270	354
100	100	307.5	290	316	404	400	420	435
		178	146	118	79.5	146		
100	100	460	451	374	365	365	285	285
98	99	215	242	214	340	320	229	182
100	100	260	234	202	285	222	227	206
100	100	125	133	151	203	139	252	192

PEEPT0	PEEPT6	PEEPT12	PEEPT24	PEEPT48	PEEPTpost2	PEEPTpost4	OuturinT0	OuturinT6
8	8	8	7	0	0	0	0	25
10	8	8	8	8	7	8	0	0
10	7	7	7	7	6	0	0	12
6	5	5	5	5	5	5	35	17.5
6	13	10	10	12			0	0
4	5	5	5	5	5	5	50	0
8	8	8	8	6	6	6	0	0
6	6	6	6	6	5	5	10	0
6	8	8	8	6	6	6	30	20
6	0	0	0	0			0	200
4	6	6	6	6	0	0	50	0
10	10	10	11	11	11	11	80	50
10	10	10	10	10	8	5	100	200
10	10	10	6	6	6	6	100	100
0	0	0	0	0	0	0	100	100
0	0	0	0	0	5	0	50	30
8	10	10	10	10	10	10	60	60
6	6	6	6	8	6	6	100	70
8	8	8	10	10	6	6	50	10
7	7	7	6	9			100	0
6	6	6	6	6	6	6	100	125
5	5	5	6				0	0
6	6	6	0	0	0	0	30	100
5	5	5	5	5			0	0
6	6	6	6	6	0	0	0	0
0	0	0	0	0	0	0	60	50
10	10	10					0	0
0	0	0	0	0	0	0	40	20
8	8	8	8	8	8	8	0	0
8	8	6	6				100	0
5	5	5	5	5	5	5	20	50
5	5	5	5	5	5	0	70	20
7	7	6	6	6	6	6	120	0
8	10	10	10	10			20	0
8	10	10	10	10	0	0	0	0
7	8	8	8	8	5	8	0	40
0	0	0	0	0	0	0	90	0
0	0	0	0	0	0	0	80	50

OuturinT12	OuturinT24	OuturinT48	OuturinTpc	OuturinTpc	CreatT0	CreatT6	CreatT12	CreatT24	
40	100	200	200	200	.81	.58	.52	.45	
0	0	10	100	100	1.44	1.73	1.4	1.24	
20	25	50	70	90	2.39	1.73	1.32	1.15	
7	7	6	90	50	2.24	1.52	1.18	.97	
0	0	0			1.28	.92	.79	.75	
10	0	20	50	150	1.85	1.43	1.17	1.01	
0	0	0	0	0	.72	.74	.61	.59	
0	0	0	0	0	.42	.47	.5	.52	
10	10	0	0	0	1.98	1.65	1.35	.98	
100	90	90			1.38	1.1	.98	.89	
60	40	0	20	20	1.99	1.79	1.63	1.19	
50	50	50	100	100	1.15	.76	.744	.62	
80	20	20	100	100	3.9	2.88	2.4	1.88	
100	200	150	100	100	.65	.41	.39	.38	
100	100	150	60	100	2.62	1.75	1.14		1
30	0	100	100	100	4.63	2.97	2.2	1.55	
60	60	20	180	200	3.35	1.92	1.07	.81	
70	30	30	200	130	4.17	3.06	1.83	1.35	
5	5	0	0	0	4.61	3.6	3.15	2.31	
0	0	0			1.33	1.06	1.1	.92	
120	120	100	190	170	2.62	1.95	1.95	2.02	
10	20				1.7	1.39	1.31	1.51	
100	50	75	75	75	2.81	1.68	1.37	.95	
0	0	0			1.3	1.33	1.27	1.09	
50	0	0	0	0	1.54	1.5	1.34	1.07	
40	50	50	50	70	2.1	.99	.96	.8	
0					.95	.95	.86		
50	30	20	10	10	1.17	.81	.6	.58	
0	0	0	0	30	4.12	2.99	2.71		2
0	0				1.4	1.4	1.33	1.31	
60	70	40	100	100	2.33	2.1	1.35	2.33	
10	50	100	200	150	1.8	1.12	1.45	1.3	
10	10	50	70	150	1.11	1.3	1.13	1.17	
10	0	0			1.59	1.1	.75	.45	
0	0	0	100	120	1.77	2.03	1.97	1.92	
40	20	40	200	150	1.89	1.49	.99	.95	
15	0	5	0	0	8.98	7.37	4.65		3
110	0	0	200	150	1.59	1.57	1.44	1.47	

CreatT48	CreatTpost	CreatTpost	AzotT0	AzotT6	AzotT12	AzotT24	AzotT48	AzotTpost2
.35	.42	.45	.17	.14	.14	.12	.03	.16
.78	1.55	1.62	1.02	1.13	.89	.77	.49	.73
.67	.64	.55	.87	.7	.64	.53	.38	.44
.63	.72	.7	.94	.7	.56	.42	.4	.84
.51			.25	.22	.21	.23	.26	
.79	1.26	1.95	.83	.69	.52	.41	.27	.66
.59	.76	.88	.12	.13	.1	.09	.1	.13
.52	.43	.43	.08	.1	.12	.11	.13	.15
.9	1.01	.24	1.43	1.11	.86	.5	.5	.51
.89			1.12	1.02	.76	.55	.55	
1.66	1	1	1.1	.93	.84	.67	.65	.5
.5	1.2	1.01	.56	.4	.36	.32	.3	.55
1.63	2.66	3.34	1.44	1.22	1.05	.87	.99	2.01
.4	.42	.51	.83	.53	.43	.4	.34	.62
.72	.78	.72	1.95	1.37	.88	.75	.3	.21
.93	1.51	2.05	1.71	1.24	.91	.63	.32	.78
.69	1.48	1.33	1.15	.75	.7	.36	.27	.82
.72	2.08	3.1	2.78	2.06	1.23	.91	.48	.66
1.51	1.36	1.22	2.2	1.78	1.56	1.12	.8	.55
.96			.7	.53	.47	.41	.34	
1.27	1.73	2.2	.52	.41	.44	.44	.33	.49
			0	.64	.56	.92		
1.56	1.63	1.72	3.71	2.31	1.77	.88	1.06	1.31
1.18			.5	.52	.48	.38	.41	
1.08	1	1	.41	.55	.34	.23	.25	.3
.8	.96	1.16	.27	.25	.24	.25	.2	.24
			.88	.88	.28			
.6	.66	.7	.55	.39	.29	.28	.2	.25
1.44	.69	.6	2.02	1.4	1.27	.95	.92	.64
			.76	.76	.78	.63		
.78	.84	1.08	1.1	1.07	.68	1.1	.34	.4
1	1.73	1.86	.76	.4	.5	.5	.5	.62
1	2.27	2.03	.64	.64	.5	.47	.4	.81
.35			.76	.53	.39	.26	.18	
1.97	2.72	3.21	.55	.76	.76	.68	.76	1.24
.78	11.26	1.38	.6	.52	.4	.42	.32	.54
1.8	1.56	1.55	2.3	1.84	1.8	.82	.72	.51
1.24	1.63	1.6	1.93	1.86	1.55	1.39	1.15	1.79

AzotTpost4	microalbT6	microalbT1	microalbT2	microalbT4	microalbTp	microalbTp	NaT0	NaT6
.18	3	2	2	2	5.3	3.6	132	139
.91				14.9	48		135	139
.44		18.6	67	49.3	14.6	25.6	141	141
.84	10.1	3.6	6.6	23.5	15.6		134	135
			621				133	138
1.06	35.9	10.6		51.1	5.1	26.7	140	135
.18	0			208			140	133
.18			532	1440			129	134
.33	282	7					137	136
	154.2		23	23			139	136
.5	90.8						128	128
.62	77.1	75	70	63	36	27	143	137
2.4	28.7	36.1	78.2	95.3	34.1	24.6	136	140
.73	19.7	15.2	11.8	5.9	6.7	14.2	147	142
.19	44.2	40.7	48	45	69.8	72	132	133
1.19	14.4	9.4	16	15.9	8.3	11.7	144	138
1.11	170	165	157	321	120	71.8	131	135
1.24	19.3			33.9		121.8	140	135
.44	244.4	3810		3990	2960		132	134
	70	19	31				131	132
.68			29.2		24.5	26.4	135	135
							143	141
1.3	177.9			82.4	193.3		129	130
							128	127
.3							138	138
.2							130	131
							136	146
.24							137	136
.52							138	138
							137	137
.61	279	243	85.3	139			144	148
.77							128	124
1.04							143	144
							142	155
1.71			3.4				138	138
.7	19.9	40.5	18.6	25.8			140	138
.41	1.033						136	138
1.62			22.3	8.6	4.6		157	152

NaT12	NaT24	NaT48	NaTpost24	NaTpost48	KT0	KT6	KT12	KT24
138	137	134	137	135	3.4	4	4	3.5
138	137	135	134	131	5	4.3	4.4	4
138	137	135	140	140	3.4	3.9	3.7	3.5
134	135	134	134	137	4	3.8	3.6	3.7
138	142	141			3.5	3.8	4.5	4.6
129	130	135	135	134	4	4.1	4.1	3.7
133	133	131	132	133	3.8	3.6	3.8	3.7
136	136	138	136	137	4.6	4.6	3.9	4.1
133	131		129	130	3.7	3.7	3.8	3.7
136	136	136			4.4	4.2	3.3	3.7
130	127	128			4.1	4.1	4	3.9
136	136	136	138	137	4.1	3.5	3.5	3.5
140	140	138	140	145	4.4	3.4	3.2	3.6
138	137	137	139	140	4.2	4	4.1	4.2
139	139	138	138	140	4.3	4.1	3.6	3.5
137	136	138	137	139	3.8	3.6	3.5	3.1
137	139	139	139	143	3.9	3.2	3.2	3.2
135	130	129	129	137	3.9	3.8	3.6	4
136	135	136	135	134	4.8	4.1	3.5	3.6
131	130	139			3.7	3.6	3.8	4.6
134	135	135	137	139	4	3.8	3.9	4
139	140				4.3	4.4	4.4	3.6
135	135	135	135	135	4.5	3.6	4	4
127	130	127			4	4	4.2	4.7
137	133	133			4.5	3.7	3.6	3.9
133	132		133	135	3.8	3.7	3.8	3.8
150					4.1	5.5	5.7	
136	132		134	134	3.8	3.6	4.2	4.3
138	136	137	137	137	4.6	4.6	4.4	4.2
137	137				3.5	3.7	4	4
135	148	138	140	139	5.4	5.4	3.9	5.5
129			127	126	4.4	3.4	3.6	
142	138		138	140	3.4	3.4	4	4.5
133	133	135			5	4.2	4.2	4.3
139	136	137	137	139	4	3.8	3.8	3.3
137	137	135	135	138	3.3	3.5	3.5	3.9
136	139	136	137	138	4.9	4.4	4.3	3.8
148	142	139	140	138	3.6	3.7	3.5	4.2

KT48	KTpost24	KTpost48	PhT0	pHT6	pHT12	pHT24	pHT48	pHTpost24
3.5	3.7	4.2	7.17	7.32	7.4	7.4	7.39	7.47
3.6	4	3.6	7.38	7.4	7.42	7.45	7.47	7.39
3.4	3.8	3.9	7.22	7.377	7.435	7.46	7.55	7.403
3.7	4.4	4	7.43	7.42	7.4	7.38	7.42	7.53
5.8			7.42	7.26	7.32	7.35	7.18	
3.7	4	4	7.2	7.42	7.45	7.49	7.4	7.4
3.9	4	4	7.38	7.36	7.36	7.37	7.36	7.4
4.3	4.2	4.3	7.4	7.43	7.46	7.42	7.39	7.36
	3.7	3.4	7.35	7.38	7.37	7.42		7.37
3.7			7.34	7.33	7.35	7.32	7.32	
4.4			7.37	7.43	7.36	7.44	7.49	
3.4	3.8	4	7.44	7.46	7.45	7.47	7.47	7.54
4.2	4	3.8	7.23	7.41	7.44	7.47	7.36	7.35
4	3.7	3.3	7.45	7.43	7.43	7.43	7.46	7.3
3.74	4	4	7.42	7.49	7.4	7.45	7.43	7.43
3.6	3.5	3.4	7.38	7.49	7.49	7.49	7.51	7.53
3.6	3.8	3.9	7.3	7.44	7.39	7.44	7.41	7.5
4.3	4.1	4.8	7.33	7.46	7.46	7.52	7.47	7.48
3.9	3.5	3.8	7.22	7.27	7.36	7.33	7.39	7.31
4.7			7.49	7.49	7.52	7.39	7.43	
4	4.3	3.9	7.32	7.35	7.32	7.35	7.35	7.35
			7.3	7.32	7.3	7.3		
4	4	4	7.38	7.38	7.38	7.38	7.38	7.38
4			7.33	7.44	7.43	7.44	7.44	
4.1			7.35	7.36	7.38	7.36	7.34	
	3.8	3.9	7.43	7.33	7.36	7.42		7.36
			7.31	7.29	7.26			
	3.6	3.5	7.39	7.35	7.32	7.37		7.37
4	3.8	4.1	7.34	7.36	7.37	7.37	7.44	7.48
			7.38	7.42	7.43	7.45		
3.7	4.1	4.3	7.41	7.38	7.41	7.49	7.48	7.56
	4.1	3.8	7.45	7.44	7.48			7.39
	3.5	3.4	7.41	7.43	7.3	7.38		7.45
4.2			7.19	7.13	7.14	7.23	7.13	
3.5	4.1	3.7	7.4	7.39	7.37	7.43	7.5	7.37
4	4	4.5	7.45	7.45	7.44	7.45	7.49	7.45
4	4	3.5	7.37	7.34	7.41	7.4	7.44	7.38
4.2	3.8	3.9	7.44	7.41	7.41	7.42	7.44	7.42

pHTpost48	HCO3T0	HCO3T6	HCO3T12	HCO3T24	HCO3T48	HCO3Tpost	HCO3Tpost	BET0
7.48	16.6	20.7	23.1	24.9	25.6	29.3	28	-10
7.52	22.9	23	24.3	35.1	34	27	27.4	-2.1
7.4	18.1	24.1	24.8	26	28	26.3	28	-7
7.47	25.1	25.7	25.2	25	26.9	24.2	29.3	.7
	19	17.9	20.6	21.1	16.3			-6.5
7.4	22	21.4	23	25	24	22	22	-4
7.4	25.9	25	26	25	25	24	23	1.7
7.4	21.7	24.5	25.7	25	24.8	26.3	22	0
7.42	28.5	26.2	25.3	25.1	23	21.2	21.7	2.6
	21.6	22.6	21.1	23.3	23.3			-3.3
	20.5	22.9	22.1	23	24.6	24	24	-4.7
7.53	29.6	29.6	30	28	20	30.1	30.7	5.7
7.35	20	24	26	26	24	24	30	-5
7.3	33.8	31.6	30.6	30.3	30	32.4	25.4	10
7.43	23	25.7	25.2	27.9	25.1	25	25	-.6
7.55	22.8	25.3	25.7	24.5	25.8	30	33.7	-2
7.48	19	22.9	23.6	23.5	23	31	39.4	-5.8
7.47	19.2	22.3	23.24	27.9	25.6	29	29.2	-6.4
7.31	20.7	19	21.6	21.2	24	22.6	23.5	-4.4
	25.1	24.6	24.9	23.3	22.9			.8
7.41	18.4	20	20	22	22	21	21	-7.5
	18.9	19.2	17	18				-6.7
7.38	25.3	24	23	23	23	23	23	1
	14.7	21.1	20	21.4	23.3			-12.5
	20.9	21	24.9	24.9	21.2	23	23	-4.2
7.45	25	24.4	24.5	25.7	24	24.5	26.7	.7
	21.9	19.9	21.5					-3
7.41	25.3	24.9	25	25.7	24	24.7	24.6	1
7.42	23.4	22.5	21.4	21.8	23.5	24.7	24.5	-1.2
	21.4	24.9	22.7	24.4				-3.6
7.52	19.8	22.3	25.1	26.9	26.3	28.2	29.6	-5.6
7.36	27.2	27.5	28	25	24	26.2	27.1	3.1
7.47	25.7	23.4	23.8	23	25	28.3	27.7	1.4
	15.9	13.9	14.7	17.9	13.9			-10.7
7.47	21	21.4	20.7	22.2	24.9	24.7	30.7	-4
7.47	21.5	22.5	23.2	24.8	25.8	37.9	35.9	-3.5
7.43	21.1	20	22.7	22.9	24.5	22.8	25.9	-4.3
7.48	24.5	24.7	24.9	23.4	23.6	24.6	26.1	.1

BET6	BET12	BET24	BET48	BETpost24	BETpost48	LatT0	LatT6	LatT12
-4.6	-1.6	.5	11.3	5.3	2.7	2.9	3.7	2.8
0	-.2	.6	2	3	3.3	1.9	2	2
-.4	.3	1.5	2.5	1.7	2	7	5.1	3.4
1.5	1	1.2	2.7	5.3	5.4	2.2	1.7	1.6
-8.1	-4.7	-3.9	-9.8			5.8	8	10.2
-3.6	-2	.4	1	1	1	3.7	6.5	5.4
1.3	2.1	1.4	.6	1	1	7	5	3
1.4	1.3	.6	.4	2.1	1.2	1.3	1.1	1
2	.9	1.1		4.1	3.8	2.1	2.3	2.4
-2.2	-4.1	-1.3	-1.3			5.2	4	2.7
-1.8	-2.8	-1.7	.2			2.4	1.7	1.4
6.3	6.4	4	2.8	6.2	6.8	1	1	1.1
.5	2	2	.2	-.5	4.6	2.9	2.9	2
7.9	6.8	6.5	6	8.6	9.6	.7	.8	.9
1.4	.8	3.8	.8	1.6	1	2.3	1.7	.7
.9	1.4	.1	1.6	6.4	10	1.4	1.8	1.7
-.5	-.9	1.2	-1.7	7.9	15.4	1.5	1.8	1.2
-2.5	-1.2	.6	1.3	5.6	5.3	1.6	1.9	1.6
-6.7	-3.3	-3.9	-.5	-2.2	-2.3	3.3	22.6	2.7
1.2	1.5	-1.4	-1.8			1.3	1.3	.9
-5.3	-4.6	-3	-3	-3.5	-3.4	2.5	2.1	2.2
-6.4	-9.2	-8				11.6	10.3	12.3
-.5	1	0	1	1	1	3	2	.5
-4	-5.5	-4	-1.4			6.7	3.2	2.4
-4.1	-1.8	-1.8	-3.9			3.6	6.3	3.7
0	.1	1.4		.3	2.6	1.3	1.1	1
-6	-3.4					17	19	25
.6	.7	1.4		.3	.1	.7	.7	.8
-2.3	-3.5	-3.1	-1.1	.3	.1	2	1.8	1.7
-.5	-1.9	-.1				2.2	3.2	2.1
-2.5	.7	2.8	2.1	4.2	5.7	13.2	8.1	3.9
3.4	4			1.9	3	.4	.9	1.3
-1.2	-.7	-1.7		4.3	3.7	4.8	4.1	3.5
-13.6	-9.7	-7.9	-13.6			18	18	17
-3.1	-4.5	-2.7	.5	-.1	6.8	4.1	3.5	3.7
-2.3	-1.4	.4	1.2	2.7	2.5	2.4	1.8	1.4
-4.4	-2.2	-1.9	.1	-1.9	2.6	2.9	5.6	2
.3	.5	-1.2	-1	.2	1.9	1.1	.8	1.3

LatT24	LatT48	LatTpost24	LatTpost48	SvO2T0	SvO2T6	SvO2T12	SvO2T24	SvO2T48
3.1	1.7	1	.7	50	65	65	77.1	76
2.1	2.5	1.3	.8	40	50	55	57	58
3.1	2.5	1.7	1	50	69.6	70	71	71
1.4	2	2.5	1.7	40	70	70	71.6	75
13.7	24			73.1	58	64.9	59	54
3.2	2	1	1	37	51	55	56	65
.8	1.5	1	1	44	57	65	82.1	81.8
.8	1	.9	.6	50	69.6	70	71	71
2.4	2.4	5.1	5.1	85.4	82.1	79.3	78.2	78
3	3			73	70	70	70	70
1.7	2	1.5	1.5	47	51	57	60	54
1.2	1.3	1.5	1.1	63	78	64	64	67
1.8	1.4	.8	1	74	78	76	67	64
.8	.7	.7	.7	65.8	67.6	73.3	76.4	70
.7	.7	.5	.5	56	79	85	75	62
1.3	1	1.5	1.6	61	71	69.5	72.5	72.3
1	1	1.7	.9	65	70	68	72	71
2	1.7	1.6	1.6	69.7	70	70	60	70
2.1	1.8	1.8	1.8	50	60	65	65	65
2	3.4			42.5	38.7	39	40.4	69.2
2	2	2.7	2.1	83.2	77.3	78.1	80	80
12				40	45	45	45	
.7	.4	.5	.5	45	56	75	72	73
2.6	2.5			45	54	55	53	56
1.9	2.2	2	2	67.2	67	66	66	62
1.2	1.1	1.1	.8	50	60	65	65	65
				59	58	58		
.7	.7	.8	.7	78.1	77.4	77.9	80	79
1.8	3.1	2.2	2.4	50	60	65	65	65
3				50	51.3	50	50	
2.4	1.6	1.8	1.9	65	70	68	72	71
1	1	.9	.6	56	79	85	75	62
3.3	2	1.3	1.3	65	70	68	72	71
18	18			42.5	38.7	39	40.4	69.2
3.6	2.6	.4	1.4	79	81	80	79	72
1.4	1.7	.8	.7	45	55	60	63	61
1.5	1.3	1.3	.9	85.4	82.1	79.3	78.2	78
1	1	.8	.8	79	81	80	79	72

SvO2Tpost	SvO2Tpost	GBT0	GBT6	GBT12	GBT24	GBT48	GBTpost24	GBTpost48
75	75	2.68	2.97	2.79	3.27	2.45	1.76	2.07
63	65	25.5	25.2	26.4	21.9	15.8	10.1	14.2
72	72	2.71	5.49	6.37	9.07	11.1	19.2	16.6
75	75	24.6	34.5	35.8	28.3	10.7	3.31	2.54
		.5	.75	1.41	3.1	3.76		
70	75	1.85	4.74	6.91	13.9	13.2	9.24	9.6
80	80	8.72	10.2	8.06	7.36	7.75	7.06	6.14
72	72	5.72	5.72	5.79	5.86	5.32	7.02	6.92
78	77	36.8	43.5	48.7	45.5	40	40.2	37.3
		30.4	29.6	28.9	22.8	22.8		
60	60	31.14	35	38	37.62	28.2	14	14
68	63.7	17.3	17.1	13.1	7.7	7	9.9	10
68	71	29.8	31.9	22.6	23.4	23.3	17.3	19
74.8	72.8	8	10.4	11.7	9.7	14	8	3.3
78	79	13.2	13.3	11.3	8.5	6.6	5.3	5.2
72.4	74.2	30.3	29.9	26	10.3	16.4	14.7	15.2
75	78	23.9	23.1	19	17.1	16	14.2	13.7
70	70	18.5	14.5	12.3	11.6	12.4	7.47	9.3
65	65	9.05	14.9	12.2	15.1	14.6	14.2	13.9
		15.7	21.4	23.4	19.6	21.8		
80	80	15.4	15.6	13.2	13.3	9.77	8.62	8.8
		14.3	15.3	13.3	12			
72	72	12	14.4	14.1	22.7	31.3	25	20
		6.85	7.86	7.38	9.58	12.5		
60	60	5.74	6.3	8.83	12.5	19.3	15	15
65	65	14.5	14	15.1	13.2	13	15.1	12.7
		12.8	20.2	21				
77.4	77.9	7.25	6.98	6.63	7.62	7	7.02	7.26
65	65	33.5	41.4	39.6	39.6	35.8	23.7	23.9
		24.6	35	30.2	60.2			
75	78	27.2	25.5	25.2	27.6	13.3	13.9	19.3
78	79	50.9	39.1	36.5	30	26	25.3	20.2
75	78	4.52	3.93	5.26	5	5	3.15	4.31
		28.7	21	14.2	3.78	1.68		
78	78	15.9	15.6	15.8	15.9	13.1	16.8	11.9
74.6	78	16.5	18.6	26.7	32.6	22.4	10.9	17.8
78	77	18.7	20.1	17.8	22.5	22.8	8.8	6.87
78	78	34	30.4	39.6	36	23.8	17.3	15.1

PltT0	PltT6	PltT12	PltT24	PltT48	PltTpost24	PltTpost48	ATIIIT0	ATIIIT6
31	30	33	35	25	23	22	30	23
239	234	252	275	266	260	252	79	64
87	78	59	56	40	84	109	57	56
205	234	198	200	144	69	72	44	36
19	16	16	18	5			35	21
45	37	39	45	32	116	133	54	42
94	101	90	91	97	39	36	54	55
17	17	31	31	31	91	109	49	49
249	246	253	260	250	250	243	28	21
270	254	254	254	254			68	48
49	58	66	56	40	45	45	71	50
289	254	245	309	307	339	373	64	60
176	184	167	199	153	129	151	43	63
91	102	78	38	28	79	71	49	52
129	148	139	117	106	98	92	91	79
86	87	85	97	45	59	70	41	23
300	296	291	257	210	140	133	119	95
409	413	423	402	312	188	216	83	71
79	92	84	102	78	63	66	59	56
99	107	102	107	47			81	70
226	196	163	167	151	153	140	27	22
48	36	27	26				63	41
158	203	174	171	132	131	120	82	86
143	136	130	105	51			27	22
67	73	66	37	10	20	20	43	52
106	112	114	117	115	114	112	70	59
67	44	30					22	10
59	62	65	65	60	53	49	83	76
220	222	204	201	162	230	180	54	54
662	592	568	562				74	51
68	94	103	68	88	73	89	46	50
56	31	69	50	50	39	63	70	68
81	62	70	69	70	79	83	50	35
16	17	14	11	9			64	41
78	103	55	43	24	32	44	83	54
255	230	237	236	159	305	343	69	58
115	126	116	146	139	104	129	84	48
445	424	278	291	208	231	271	75	66

ATIIIT12	ATIIIT24	ATIIIT48	ATIIITpost2	ATIIITpost4	PCTT0	PCTT6	PCTT12	PCTT24
87	44	81	61	68	29.33	40.31	43.08	25.28
68	59	56	58	68	2.93	3.57	5.07	5.08
47	50	29	44	42	20.38	16.32	17.75	17.16
34	30	32	48	40	9.92	7.22	6.17	5.47
20	41	19			19.76	18.64	23.5	14.44
67	43	36	71	84		11.73	13.79	16.96
52	54	56	96	90	.77	.6	.54	.22
41	44	54	48	57	1.76	1.41	1.18	.91
15	13	13	11	10	4.52	2.69	2.15	2.12
40	40	40			7.18	5.81	5.08	4.6
43	71	54	55	55	2.39	1.21	1.21	.62
51	63	60	95	81	.5	.5	.5	.5
57	55	71	88	118	136.5	103.8	80.5	73
46	55	95	102	106	5	1.6	1.4	1
58	57	63	77	95	54	50.7	43.2	31.2
33	23	69	94	73	200	125	93	59.4
92	85	78	104	106	2.2	2.2	2.2	2
57	61	91	85	70	6.42	4.25	2.8	1.6
38	45	50	67	99	20.56	10.9	10.09	7.4
69	70	70			5.58	3.45	3.13	2.87
22	26	27	29	36	3.61	3.29	2.52	2.21
29	28				6.71	5.5	4.87	4.42
88	89	86	107	100	.73	.52	.43	.28
22	40	45			1.75	1.81	1.36	1.37
48	51	53	50	60	20.56	10.9	10.09	7.4
60	55	55	60	67	.1	.13	.1	.9
6					6.12	5.09	2.5	
66	68	65	70	68	.23	.11	.18	.2
54	51	50	59	56	.93	.93	.58	.63
38	38				.15	25	80	90
35	46	32	44	51	54.55	33.89	33.1	54.55
80	77	78	87	96	2.04	1.45	1.43	1.1
28	28	25	25	22	2.04	1.45	1.43	1.1
31	20	7			7.28	8.53	2.26	1.65
44	67	53	73	71	19.9	14.35	13.79	12.16
59	75	78	75	78	134.22	60	48	35
48	58	48	84	87	49.83	37	44	36
49	45	53	68	58	2.07	1.8	1.63	2.01

PCTT48	PCTTpost2	PCTTpost4	PCRT0	PCRT6	PCRT12	PCRT24	PCRT48	PCRTpost2
50	8.31	4.45	79	105	120	144	128	57
2.18	1.44	1.28	306	352	339	356	206	259
8.35	3	1.91	276	322	382	443	80.35	168
2.28	.37	.26	206	282	292	309	175	27
7			135	143	150	217	205	
12.1	.52	.45	212	264	307	401	377	52
.13	.14	.16	150	160	161	144	130	83
.6	.34	.38	136	115	116	102	84	42
2	1.7	1.63	104	92	91	85	70	48
4.6			150	296	395	407	407	
.66	.5	.5	48	50	45	52	49	35
.5	.4	.3	23.51	26.16	25.53	19.64	16.5	8.21
54.4	60.5	27.7	36.6	37.6	39.65	33.66	18.87	5.1
.2	.1	.1	5.42	5.43	6.43	6.93	6.35	1.77
17.08	4.31	1.91	9.04	10.8	10.5	8.5	6.3	2.34
42.2	22.5	15.1	24.88	21.88	15.96	9.02	8.64	4.9
2.5	3.3	1.1	16.26	15.18	14	14.75	11.03	7.11
.15	.42	.35	45	48	85	97	114	101
7.98	1.6	1.39	364	641	303	341	274	221
1.5			237	231	229	179	116	
1.61	1.94	1.69	268	266	236	247	202	144
			71	70	71	62		
.83	.69	.6	9	9	9	9	50	50
1			161	160	154	124	83	
7.98	1.6	1.39	248	201	201	101	96	71
.8	.1	.09	65	42	20	23	20	40
			80	80	96			
.1	.18	.15	248	201	201	101	96	71
.84	6.95	4.22	150	150	160	161	166	76
			82	55	43	28		
17.27	14.71	11.22	237	300	311	237	151	58
1.1	1.42	1.12	300	127	112	80	80	85
1.1	1.42	1.12	136	115	116	102	84	42
1.1			212	201	198	169	181	
7.3	1.86	1.86	85	78	83	67	31	9
28	2.62	2.6	248	201	201	101	96	71
27	6.39	3.36	237	237	294	348	311	170
2.31	6.1	3.82	179	172	160	152	157	146

PCRTpost4	Klebsiella	Pseudomo	Escherichia	Aureus	Pneumoco	Candide	Asperigilli	Altro
60	0	0	0	0	0	0	0	1
304	0	0	0	0	0	0	0	1
135	0	0	0	0	0	0	0	1
20	0	0	0	0	1	0	0	0
	1	0	0	0	0	1	0	0
34	0	0	0	0	1	0	0	0
78	1	0	0	0	0	0	1	0
42	0	0	1	0	0	1	0	0
37	0	0	0	0	1	0	0	0
	0	1	0	0	1	0	1	1
35	0	0	0	0	0	1	0	0
9.29	0	1	0	0	0	0	0	0
4.17	1	0	0	0	0	0	0	0
.98	0	0	1	0	0	1	0	1
.91	0	1	0	0	0	0	0	0
3.5	0	0	0	1	0	0	0	0
9.74	0	1	0	0	0	0	1	0
78	0	0	1	0	0	1	0	0
139	0	0	0	0	0	0	0	1
	1	0	0	0	0	0	0	0
93	0	0	0	1	0	0	0	0
	0	1	0	0	0	0	0	0
45	0	0	0	1	0	0	1	1
	0	0	1	0	0	0	0	0
89	0	1	0	0	0	0	0	0
28	1	0	0	0	0	0	0	0
	0	0	0	1	0	0	0	0
89	0	0	0	1	0	0	0	0
27	0	1	0	0	0	0	0	0
	0	0	1	0	0	1	0	0
110	1	0	0	0	0	0	0	0
51	0	0	1	0	0	0	0	0
42	0	1	0	0	0	1	0	0
	0	0	0	1	0	0	0	0
14	0	0	1	0	0	0	0	0
89	1	0	0	1	0	1	0	0
61	0	0	1	0	0	0	0	0
139	1	0	0	1	0	0	0	0
